# Understanding the molecular regulatory mechanisms of autophagy in lung disease pathogenesis

**DOI:** 10.3389/fimmu.2024.1460023

**Published:** 2024-10-31

**Authors:** Lin Lin, Yumeng Lin, Zhongyu Han, Ke Wang, Shuwei Zhou, Zhanzhan Wang, Siyu Wang, Haoran Chen

**Affiliations:** ^1^ School of Medical and Life Sciences, Chengdu University of Traditional Chinese Medicine, Chengdu, China; ^2^ Nanjing Tongren Hospital, School of Medicine, Southeast University, Nanjing, China; ^3^ School of Medicine, Southeast University, Nanjing, China; ^4^ Science Education Department, Chengdu Xinhua Hospital Affiliated to North Sichuan Medical College, Chengdu, China; ^5^ Department of Science and Education, Deyang Hospital Affiliated Hospital of Chengdu University of Traditional Chinese Medicine, Deyang, China; ^6^ Department of Radiology, Zhongda Hospital, Nurturing Center of Jiangsu Province for State Laboratory of AI Imaging & Interventional Radiology, School of Medicine, Southeast University, Nanjing, China; ^7^ Department of Respiratory and Critical Care Medicine, The First People’s Hospital of Lianyungang, Lianyungang, China; ^8^ Department of Preventive Medicine, Kunshan Hospital of Chinese Medicine, Kunshan, China

**Keywords:** autophagy, pulmonary diseases, apoptosis, autophagosome, COPD

## Abstract

Lung disease development involves multiple cellular processes, including inflammation, cell death, and proliferation. Research increasingly indicates that autophagy and its regulatory proteins can influence inflammation, programmed cell death, cell proliferation, and innate immune responses. Autophagy plays a vital role in the maintenance of homeostasis and the adaptation of eukaryotic cells to stress by enabling the chelation, transport, and degradation of subcellular components, including proteins and organelles. This process is essential for sustaining cellular balance and ensuring the health of the mitochondrial population. Recent studies have begun to explore the connection between autophagy and the development of different lung diseases. This article reviews the latest findings on the molecular regulatory mechanisms of autophagy in lung diseases, with an emphasis on potential targeted therapies for autophagy.

## Introduction

Pulmonary diseases, especially chronic pulmonary diseases, including chronic obstructive pulmonary disease (COPD), pulmonary tuberculosis (PTB), and lung cancer, pose significant threats to human health. Despite notable advancements in research globally in recent years, effective and precise treatments are still insufficient, leaving many lung diseases without a cure.

Autophagy is a common phenomenon in eukaryotic cells that fuses with lysosomes and hydrolyzes intramembrane components by encasing damaged or functionally degenerated organelles and certain proteins and certain macromolecules. Autophagy was first identified in the 1850s and named in 1963 by de Duve et al (1). Recent research has indicated that autophagy is important for maintaining cellular survival and homeostasis (2–4). Through the processing of metabolic precursors from cytoplasmic substrates, this process maintains homeostasis in healthy respiratory cells and ensures survival in conditions of nutrient scarcity (5). In nutrient deficiency, cells acquire nutrients through autophagy; damaged or senescent organelles can be removed by autophagy when cells are damaged or senescent; and these microorganisms or toxins can be cleared by autophagy when cells are infected by microorganisms or invaded by toxins. Eukaryotes have well-preserved degradation and recycling processes critical to maintaining cellular homeostasis and coping with stress. To some extent, autophagy is an effective mechanism to protect cells.

**Figure 1:**
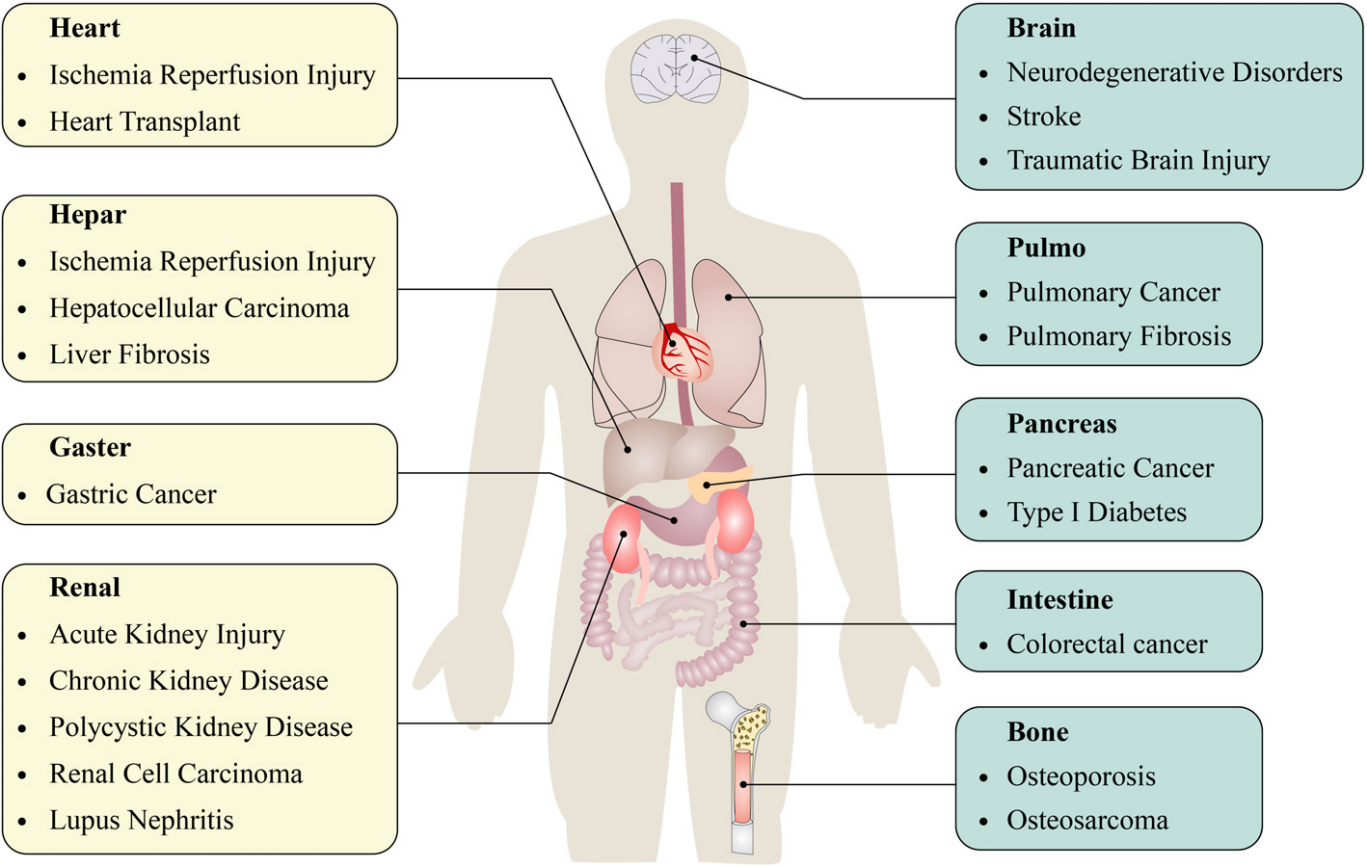
Autophagy is involved in the development and progression of multiple diseases.

**Figure 2:**
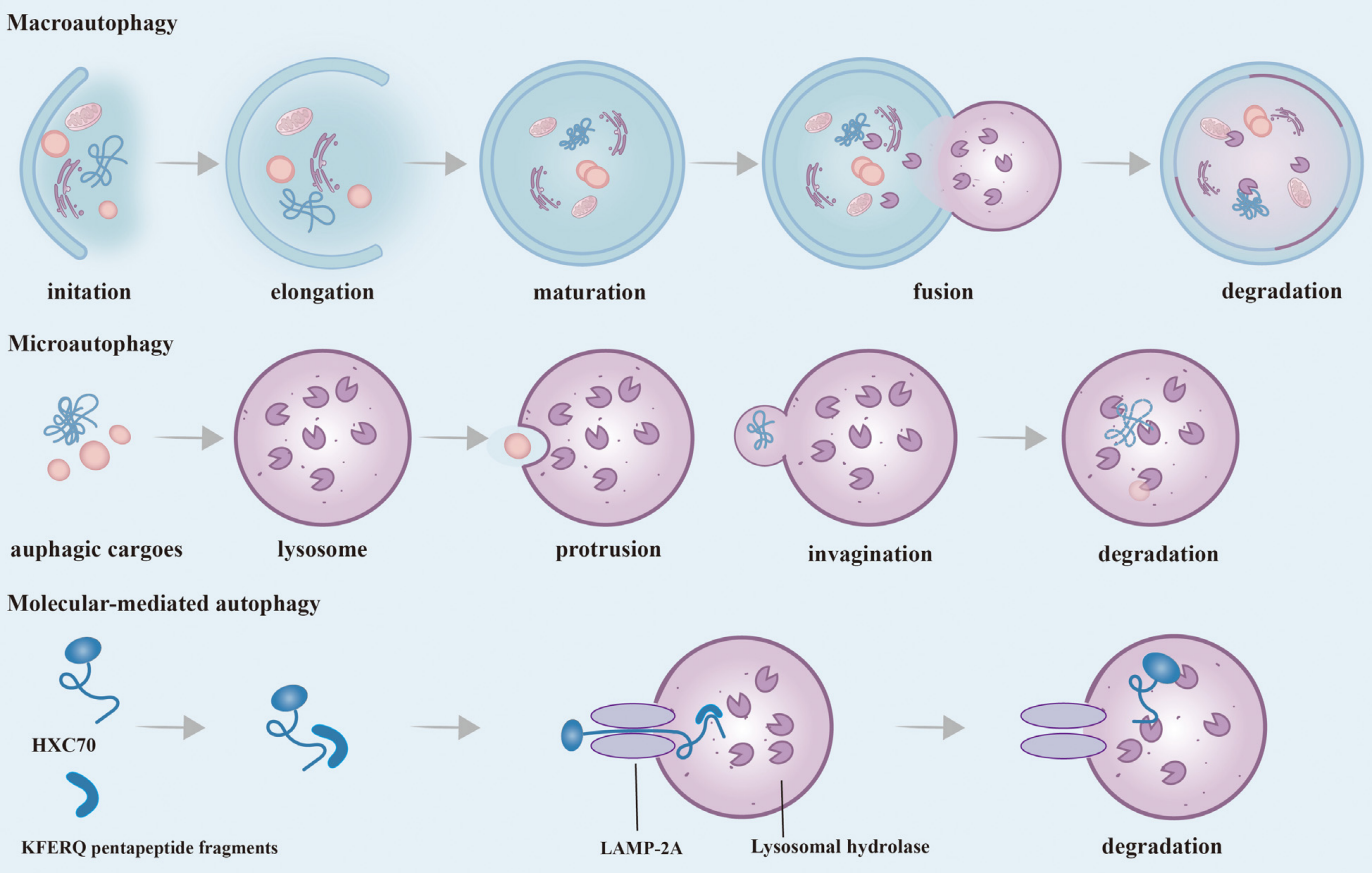
Phases and Classification of Autophagy. According to the different ways of transporting substrates to lysosomes, autophagy can be divided into three main ways: macroautophagy, microautophagy and CMA. Macroautophagy: It starts as autophagy-related substances accumulate around misfolded and aggregated proteins, pathogens, non-essential amino acids, etc. to form a barrier membrane. Dysfunctional organelles as well as proteins are surrounded by an isolation membrane and gradually form a bilayer membrane structure, called autophagosomes. The outer membrane of autophagosomes then fuses with lysosomes, and internal material is degraded in autolysosomes. Microautophagy: The process by which membranes of lysosomes encapsulate cargo by direct protrusion or invagination and are degraded in lysosomes. CMA: Substrate proteins containing the KFERQ-like pentapeptide sequence are first recognized by HSC70, then bind to LAMP-2A on the lysosomal membrane and enter the lysosome and eventually are degraded. CMA, Chaperone-mediated autophagy; HSC70, Heat shock cognate protein 70; LAMP-2A, Lysosomal membrane-associated protein 2A.

Autophagy is intricately associated with the clearance of organelles and, more significantly, plays a crucial part in the development and progression of various diseases. The relationship between autophagy and disease pathogenesis has not been fully confirmed. Nonetheless, a growing body of evidence indicates that autophagy may play a significant role in various human diseases (2, 6), including inflammatory diseases (7–9), cardiovascular diseases (10, 11), neurodegenerative diseases (12), and cancer (13) (**Figure 1**). Alterations in autophagic activities may also result from variations in the activation of proteins that regulate autophagy (2, 14). Until now, only limited studies have investigated the role of autophagy in lung disease **Figure 2**.

This review highlights the most recent developments in the molecular control and the role of autophagy in lung diseases. Additionally, we explore how autophagy-related proteins and regulatory processes may contribute to either the protection against or the advancement of human lung diseases, offering new insights for targeted treatment options.

## Phases and classification of autophagy

Autophagy is essential for the process of protein degradation with relatively short half-lives. Morphologically, a significant quantity of dissociative membranous structures appears in the cytoplasm of cells that are about to undergo autophagy, which are called proautophagosomes. The proautophagosome gradually develops into a vacuole with a double membrane structure, which is surrounded by degraded organelles and some cytoplasm (2, 15). This double membrane structure is referred to as the autophagosome (2). Next, after autophagosomes fuse with lysosomes, the inner membranes and their encapsulated substances enter the lysosome and undergo hydrolysis by lysosome enzymes. The lysosomes found in this phagocyte are called autolysosomes. This process leads to the retrieval of soluble cytoplasmic proteins, mitochondria, peroxides, Golgi complexes, and portions of the endoplasmic reticulum, while some digested fragments are released into the cytoplasm for biosynthesis (3, 5, 16).

According to the different ways of transporting substrates to lysosomes, autophagy can be divided into three main ways: macroautophagy, microautophagy, and chaperone-mediated autophagy (CMA) (17). Macroautophagy is the most common autophagy in eukaryotic cells by forming a double-layer membrane around misfolded and aggregated protein pathogens, and non-essential amino acids, and fusing with lysosomes for degradation. Many stresses, such as nutritional deficiency, infection, oxidative stress, and toxic stimulation, can stimulate the occurrence of macroautophagy, which is generally referred to as autophagy. Different from macroautophagy, there is no formation process of autophagy membrane in microautophagy. A characteristic aspect is that the lysosome membrane is straight taken in by lysosomes and late endosomes via membrane protrusion and invagination, and it is then broken down within the endolysosomal lumen. During the dependent multivesicular body (MVB) formation, a significant quantity of cytoplasmic proteins is selectively integrated into the lumens of endosomes in substantial amounts (18). CMA represents a highly selective mechanism of autophagy with two core members: the heat shock cognate protein 70 (HSC70) and the lysosomal membrane-associated protein 2A(LAMP-2A). HSC70 is a molecular chaperone protein. The process of CMA degrades proteins that contain KFERQ pentapeptide fragments in the peptide chain. First, the heat shock protein HSC70 specifically recognizes and binds to proteins containing KFERQ five-peptide fragments, and transports the target protein into the lysosome for degradation through interaction with LAMP2A (17). Macroautophagy is considered to be the predominant form of autophagy compared to microautophagy and molecular-mediated autophagy, and this has also been the subject of extensive research. Therefore, what we usually call “autophagy” is macroautophage.

In addition, autophagy can be classified into selective autophagy, aggregative autophagy, and xenophagy, etc. Recent research has demonstrated that several denatured proteins, organelles, and certain bacteria can be selectively destroyed by autophagy. This process is called “selective autophagy”, the most representative of which is mitophagy (17, 19, 20). Mitophagy is a specific degradation targeting depolarized mitochondria (21). Xenophagy involves the digestion of extracellular components containing pathogens or bacteria that invade the body (22).

## Molecular involvement in autophagy and molecular regulation

The process of autophagy is modulated and governed by various relative proteins. In mammalian cells, starvation-induced autophagy is regulated by approximately 20 core Atg genes (23). These gene products are persistently incorporated into vacuoles and assembled to construct pre-autophagosomes. In addition, the modification of microtubule-associated protein-1 light chain 3 (LC3) is an important step in forming autophagic vacuole. In autophagosomes, LC3 and its homologues act on autophagic substrates or proteins to facilitate the selection of autophagic cargoes (24).

**Figure 3:**
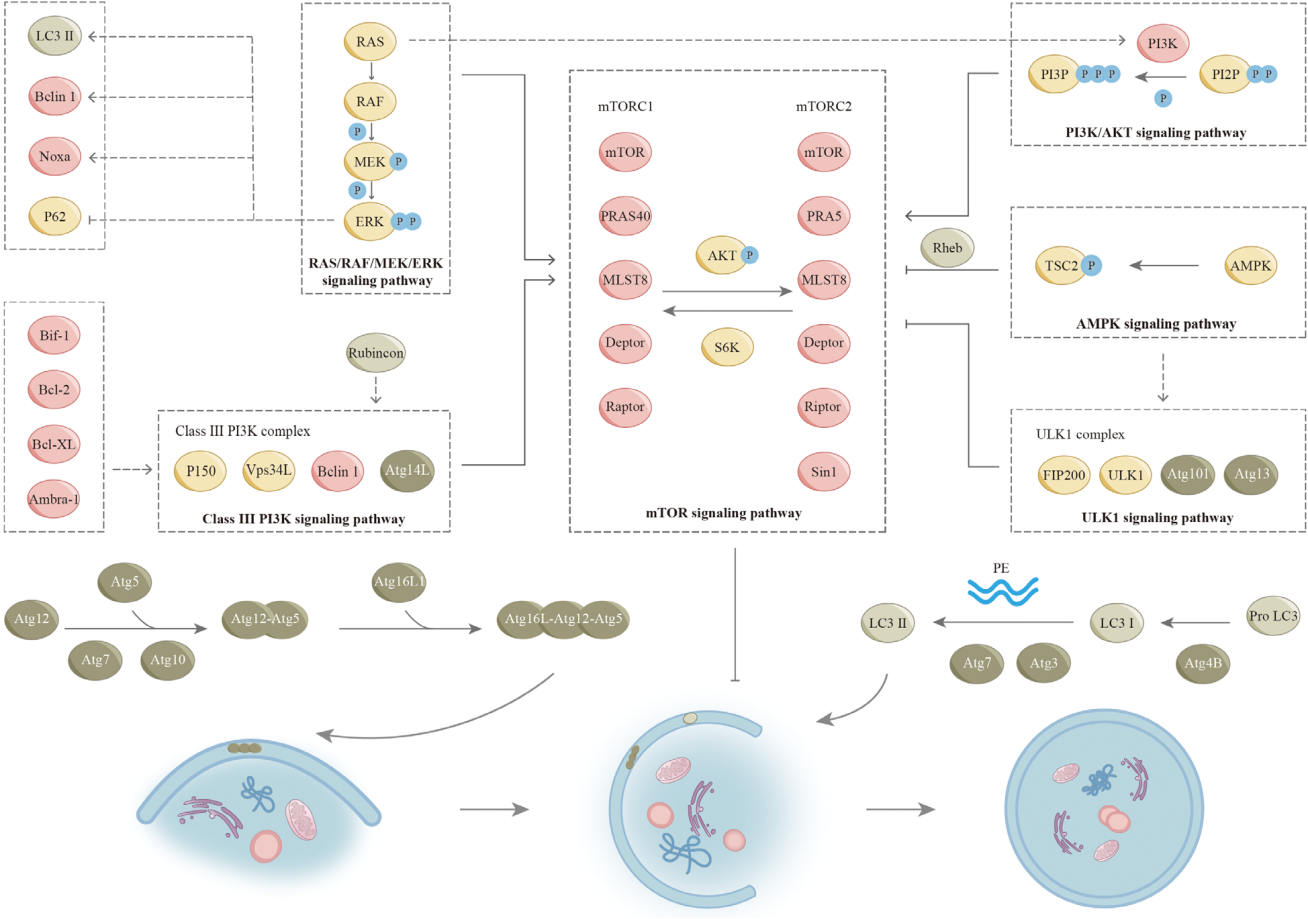
Signaling pathways for autophagy. The process of autophagy is regulated by many signaling pathways (as shown), and there is also complex crosstalk between various pathways. Two ubiquitin-like conjugation systems involved in the formation of autophagosome: In the first system, the ubiquitin-like protein Atg12 is enzymatically coupled to Atg5 by Atg7 (E1 ubiquitin-activating enzyme-like) and Atg10 (E2 ubiquitin-conjugating enzyme-like) to produce the Atg5-Atg12 complex. The Atg5-Atg12 complex interacts with Atg16L1 to form a complex that plays a role in the formation of autophagic membranes. As part of the maturation process, these factors are separated from autophagosomes. The second coupling system requires the ubiquitin-like protein LC3. LC3 and its homologues, including the isozymes of LC3 and associated proteins (e.g., GABARAP), are modified by cellular lipid PE. An important regulatory step in the formation of autophagosomes is the transformation of LC3-I (free form) to LC3-II (PE conjugated form). The precursor form of LC3 is cleaved by the protease ATG4B to yield LC3- I (not shown). ATG7 and ATG3 participate in conjugating PE with LC3-I to LC3-II. LC3-II cytoplasmic redistribution, characterized by punctate LC3 staining, is indicative of autophagosome formation. GABARAP, (GABA type A receptor-associated protein); LC3, (Microtubule-associated protein 1 light chain 3; PE, (Phosphatidylethanolamine).

The elongation stage of autophagosome formation relies on two ubiquitin-like conjugation systems (**Figure 3**), (2, 3). Besides the proteins mentioned in **Figure 3**, the maturation and fusion of autophagosomes also depend on various other proteins, such as small GTPases (like Rab7), class C Vps proteins, ultraviolet radioresistance-associated gene protein (UVRAG), and lysosome-associated membrane proteins (for instance, LAMP-2A) (25, 26). In recent years, additional proteins associated with autophagy have been progressively identified alongside the aforementioned proteins. In a complicated regulatory network, these proteins regulate the initiation and execution of autophagy (2, 27). We will describe this in detail in the following paragraphs (**Figure 3**).

## Mammalian target of rapamycin signaling pathway

Many studies have demonstrated that mTOR negatively regulates autophagy in nutrient-rich environments (28). mTOR is an atypical serine/threonine protein kinase that is evolutionarily relatively conserved. Cell cycle regulation, proliferation, differentiation, motility, and invasion are among its physiological functions. Two unique complexes, mTORC1 and mTORC2, exist within the cell and are distinguished by distinct components. mTORC1 and mTORC2 are two signaling complexes that play a major role in the mTOR pathway. Ribosomal protein S6 kinase (S6K) and kinase B (AKT) are key enzymes in the interaction between mTORC1 and mTORC2. S6K is activated by mTORC1, which subsequently activates mTORC2. Conversely, mTORC2 facilitates the phosphorylation of AKT, leading to the activation of mTORC1. mTORC1 is responsive to energy levels and stress, and it is significantly inhibited by rapamycin. A substantial body of research has indicated that mTORC1 exerts an inhibitory influence on the process of autophagy (29, 30). Unlike mTORC1, mTORC2 is not susceptible to both rapamycin and nutrients because of the presence of Rictor (31). However, long-term rapamycin treatment ultimately inhibits mTORC2 activity (32). mTOR is a key molecule during autophagy induction. Many signaling pathways have the capacity to either promote or inhibit the process of autophagy through their interactions with mTOR (**Figure 3**). Nonetheless, the enhancement or suppression of autophagy by these pathways is not definitive. In some specific cases, the opposite effect may also be exerted. We will describe it further in the following sections.

mTOR is integral to numerous physiological functions, such as cell proliferation, survival, and autophagy, which is intricately linked to various lung diseases through its regulatory effects on cell growth, inflammation, and fibrosis. We will describe it in the following sections. The mTOR signaling pathway plays a critical role in the development and maintenance of lung function. It regulates the growth and differentiation of lung epithelial cells and fibroblasts to maintain normal lung function. Moreover, mTOR signaling is involved in the immune response to pulmonary pathogens, which regulates the activation of immune cells and the inflammatory response. Given its central role in lung diseases, mTOR signaling has become a target for therapeutic intervention.

## The phosphoinositide-3-kinase protein/kinase B signaling pathway

PI3K/AKT was discovered in the 1980s and plays an important role in major physiological activities of cells (33). PI3K-AKT signaling mainly involves two metabolites, phosphatidylinositol-4,5-bisphosphate (PIP2) and phosphatidylinositol-3,4,5-bisphosphate (PIP3), and two coding genes, lipid phosphatase (PTEN) and 3-phosphoinositide-dependent protein kinase-1 (PDK1). PIP2 is converted to PIP3 by phosphorylation in response to PI3K. Next, PIP3 on autophagosome membranes recruits ATG18 and binds to bilayer membranes, allowing autophagosomes to extend and complete (34). PDK1 is a key regulatory molecule of the PI3K-AKT signal transduction pathway and plays an important part in the activation of AKT (35–37). In addition, mTORC2 can directly activate AKT by phosphorylating Ser-473 (36). PTEN is an important negative regulator of PIP2 conversion to PIP3. PTEN acts to promote dephosphorylation of PIP3 thereby inhibiting its accumulation in cells (38). Once activated, AKT acts on various cytoplasmic proteins to mediate cell growth and survival. The main downstream effector is mTOR. Furthermore, AKT influences the interaction between phosphorylated tuberous sclerosis complex 1 (TSC1) and phosphorylated tuberous sclerosis complex 2 (TSC2), consequently facilitating the activation of mTORC1 via the H-Ras-like GTPase (Rheb) (39). Subsequently, active mTORC1 inhibits autophagy by blocking the uncoordinated 51-like protein kinase (ULK1) (40).

The PI3K/AKT signaling has a tight relationship in regulating cell growth, survival, and metabolism. This pathway is involved in numerous cellular processes and has significant implications for various lung diseases. In certain pathological conditions, the PI3K/AKT signaling pathway is frequently activated in reaction to inflammatory stimuli and oxidative stress, which results in airway remodeling and contributes to the pathophysiology of the disease, including mucus hypersecretion and smooth muscle cell proliferation, therefore enhancing the survival of inflammatory cells in the lungs. The PI3K/AKT pathway presents multiple potential therapeutic targets for treating lung diseases. Inhibitors of PI3K, AKT, or associated pathways are currently undergoing investigation to reduce inflammation, fibrosis, and tumor growth in lung diseases.

## RAS/RAF/MEK/ERK signaling pathway

As a significant signaling pathway of mitogen-activated protein kinase (MAPK), RAS/RAF/MEK/ERK is involved in regulating cell proliferation, differentiation, apoptosis, and numerous signaling pathways (41, 42). RAS is a small GTPase that is activated by several factors, including receptor tyrosine kinases, growth factors, heterotrimeric G proteins, integrins, serpentine receptors, and cytokine receptors. Furthermore, oxidative stress activates the RAS/RAF/MEK/ERK signaling pathway. Notably, certain growth receptors are not required for ROS-induced RAS activation (43). In addition, ROS can uncouple MAPK pathway activity from RAS expression (44). Activated RAS further recruits RAF (MAPKKK) to the plasma membrane for activation. Following this, RAF activates and phosphorylates MEK (MAPKK), followed by ERK (MAPK). As ERK is activated, it translocates to the nucleus and triggers transcription and expression of target genes (45, 46). The expression products of these genes regulate various physiological functions of cells, including the regulation of autophagy (45, 46). PI3K and TSC2 are regulated by the RAS/RAF/MEK/ERK pathway, thereby activating mTORC1 activity. In addition, the activated RAS/RAF/MEK/ERK signaling pathway up-regulates LC3, Beclin1, and Noxa, and directly down-regulates p62 to induce autophagy (47, 48). Following induction by lindane, the formation of autophagosomes within cells is closely linked to the prolonged activation of ERK (49). Notably, this phenomenon occurs independently of both mTOR and p38 (49). These seemingly contradictory findings indicate that specific environmental conditions may directly influence the regulation of autophagy via the RAS/RAF/MEK/ERK signaling pathway.

Dysregulation of this pathway has been implicated in various diseases, including lung cancer. Mutations in genes encoding components of this pathway, such as KRAS, BRAF, and MEK, are commonly found in lung cancer patients. Mutations in BRAF and MEK are also observed in a subset of lung cancer patients. Furthermore, aberrant activation of the RAS/RAF/MEK/ERK pathway has been linked to other lung diseases, such as pulmonary fibrosis and COPD. In these conditions, dysregulated signaling through this pathway can lead to inflammation, tissue remodeling, and fibrosis in the lungs.

## Adenosine 5’monophosphate-activated protein kinase signaling pathway

AMPK is recognized as one of the primary substrates of LKB1 (liver kinase B1), which functions as an intrinsic energy sensor and regulator of cellular homeostasis (50, 51). AMPK is a heterotrimeric serine/threonine kinase that consists of a catalytic αsubunit and two regulatory subunits, which are β and γ. The activation of AMPK occurs in reaction to elevated levels of intracellular AMP and reduced levels of ATP, particularly during conditions of nutrients. LKB1 implements the involvement of this process by phosphorylating the α-activating loop (52). The activation of AMPK affects multiple processes, including mTOR pathway regulation and p53 phosphorylation (53). Further, AMPK is capable of directly phosphorylating Raptor or TSC2. Next, TSC2 signals to inhibit mTOC1 activity (44, 45, 54). In this pathway, AMPK negatively regulates mTORC1 by adenosine 5’ -monophosphate levels, thereby positively regulating autophagy upon energy depletion (55). Research indicates that AMPK exerts direct regulation over ULK1 in a manner that is sensitive to nutrient availability, thereby contributing to the intricate nature of regulatory mechanisms, as elaborated upon in the subsequent sections (56–58).

AMPK is a dominant y regulator of cellular energy metabolism and plays a crucial role in maintaining cellular homeostasis. Within the realm of pulmonary disorders, AMPK signaling has been demonstrated to exhibit both preventive and pathogenic effects. In several lung diseases, including COPD, asthma, and pulmonary fibrosis, dysregulation of AMPK signaling has been implicated. However, the relationship between AMPK signaling and lung disease is complex and disease-specific. Additional investigation is necessary to elucidate the specific mechanisms by which AMPK influences lung function, as well as to assess the feasibility of marking this pathway for therapeutic strategies.

## Uncoordinated-51-like protein kinase signaling pathway

ULK1 is a master regulator of autophagy initiation among mTORC1 downstream regulatory targets (59). Among the components of autophagy, ATG1, ATG13, and ATG17 are critical regulators of autophagy initiation (44, 60–62). In mammals, ULK1 and ULK2 are homologues of ATG1, and mATG13 and 200 kDa adhesion kinase family interacting protein (FIP200) are homologues of ATG13 and ATG17, respectively (59, 63–66). The importance of ULK1 in the autophagy pathway is reflected in its involvement in forming mTOR substrate complexes (60, 66, 67). mTORC1 has been reported to inhibit its pre-autophagic effect by phosphorylating ULK1 under normal and nutrient-rich conditions (68). mTORC1 is also able to directly phosphorylate and inhibit ATG13, one of the activators of ULK1. ULK1 can activate autophagy by phosphorylating Beclin-1 indirectly involved in the formation of VPS34-Beclin-1-ATG14 (29, 69, 70). In addition, AMPK can directly interact with ULK1 to regulate ULK1 in a nutrient-sensitive manner. Activating ULK1 by phosphorylating Ser 317/Ser 777, AMPK acts by triggering autophagy in response to glucose and amino acid starvation (58). Interestingly, mTORC1 blocks the cellular collection between ULK1 and AMPK by phosphorylating Ser 757. Consequently, it can be inferred that ULK1 equips cells with the capacity to effectively respond to intricate environmental alterations in conjunction with mTORC1 and AMPK.

Recent studies denote that ULK1 signaling may be implicated in the development of several pulmonary disorders. In conditions like IPF, COPD, and lung cancer, dysregulation of autophagy, including ULK1 signaling, has been implicated in disease progression. Investigating the function of ULK1 signaling in lung diseases may facilitate the creation of targeted therapies designed to regulate autophagy and improve outcomes for patients with these conditions.

## Type III phosphatidylinositol triphosphate kinase signaling pathway

Autophagosome formation is closely dependent on class III PI3K complexes. Activated class III PI3K complexes lead to increased PI3P formation, and PI3P-recruiting protein factors initiate autophagosome formation, including WD repeat protein interacting with inosine phosphate (WIPI-1/2), Atg18, and protein 1 containing double FYVE (DFCP1) (71, 72). Class III PI3K complexes exist in two distinct types in mammalian cells, where complexes consisting of VPS34L, p150, Beclin1, and ATG14L are closely associated with autophagy. We refer here to this complex collectively as the class III PI3K complex. Beclin 1 serves as a significant regulator of autophagy. It is also defined as a tumor suppressor protein, exhibiting the capacity to engage with a wide variety of proteins, including ATG14L, ultraviolet resistance-associated gene protein (UVRAG), Rubicon, and Bcl-2 (73–76). Three domains play important roles in Beclin1 function, including the Bcl-2 homology 3 (BH3) domain and the central coiled-coil domain (CCD) that mediates interactions with ATG14L and UVRAG (77–80). In addition, the active ULK1 results in the recruitment of class III PI3K complexes to autophagosomes, forming alternating Beclin 1-Vps34L complexes with UVRAG and promoting autophagy (75, 81–83). Evolutionarily conserved domain (ECD) mediates communication between Beclin 1 and VPS34, which in turn activates VPS34 kinase to regulate autophagosome formation. Furthermore, class III PI3K complexes can negatively regulate autophagy in response to the newly identified factor Rubicon (84, 85). Ambra-1, Bif-1, Bcl-2, and Bcl-XL can also act on class III PI3K complexes to modulate their activity (76, 86, 87).

In the context of lung disease, dysregulation of Class III PI3K signaling has been implicated in various respiratory conditions. In diseases like IPF, COPD, and lung cancer, altered Class III PI3K signaling has been associated with disease pathogenesis and progression. Understanding the role of Class III PI3K signaling in lung diseases is important for identifying potential therapeutic targets and developing targeted interventions to modulate this pathway for the treatment of respiratory disorders.

## Wild-type p53 signaling pathway

p53 functions as a tumor suppressor protein and serves as a transcription factor that regulates gene networks in response to various cellular stresses, thereby maintaining genome stability and integrity. However, p53 not only prevents tumorigenesis but also plays a critical regulatory part in primary signaling and metabolic adaptation (88, 89). The effect of wild-type p53 on autophagy is complex, highly dependent on the environment, and determined by the cellular microenvironment and stressful conditions. The progression of the cell cycle and the subcellular localization of p53 serve dual functions in the regulation of autophagy. The dual effect of wild-type p53 on autophagy is reflected in its transcriptional activity against a range of downstream target genes with autophagic regulatory effects. The dual role of p53 in autophagy is presented in **Table 1.**

**Table 1:** The dual effects of wild-type p53 on autophagy.

Effect	Signaling pathway	Mechanism	References
Promoted	mTOR	Wild-type p53 uses AICAR to stimulate AMPK activity to inhibit key downstream effectors of mTOR signaling, such as phosphorylation of 4E-BP1 and RPS6. p53 can induce secretion of IGF-BP3 and indirectly affect the autophagic process regulated by IGF (s).	(256, 257)
Promoted	AMPK	Wild-type p53 stimulates signaling through AMPK to the β1/β2 subunit (Sestrin1/2).	(258, 259)
Promoted	DAPK-1	DAPK-1 promotes Wild-type p53 accumulation in an ARF-dependent manner, followed by stimulation of autophagy through ARF.	(260, 261)
Promoted	Bcl-2 protein family	Wild-type p53 activates multiple pro-apoptotic protein production, including Bax as well as the BH3-only proteins Bad, Bnip3, and Puma.	(262, 263)
Promoted	PI3K	Wild-type p53 in the nucleus is able to up-regulate PTEN through a transcription-dependent pathway, which in turn inhibits the PI3K pathway.	(264, 265)
Promoted	HIF-1	HIF-1 can stabilize p53, which in turn promotes the autophagic process.	(266, 267)
Promoted	ULK1	In response to DNA damage, p53 upregulates ULK1 and ULK2 expression.	(268)
Promoted	HSF-1	Wild-type p53 is involved in the induction of (Isg20L1 and HSF1), which in turn transactivates autophagy-related genes (ATG7).	(269)
Promoted	TGM2	Wild-type p53 promotes autophagic flux by enhancing autophagic protein degradation and autophagosome clearance by inducing TGM2	(270)
Inhibited	mTOR	Wild-type p53 inhibits AMP-dependent kinases, thereby activating mTOR	(271)
Inhibited	TIGAR	Wild-type p53 induced TIGAR (TP53-induced glycolytic and apoptotic modulator) regulates glycolysis and cellular ROS levels	(272)
Inhibited	miR-34a series and miR-34a/34c-5p	Wild-type p53 impacts transcriptional regulation of microRNAs (miR-34a series and miR-34a/34c-5p, against ATG9A and ATG4B, respectively)	(273, 274)
Inhibited	Beclin-1	Wild-type p53 interacts with Beclin-1 and subsequently promotes its ubiquitination and proteasome-mediated degradation	(275)
Inhibited	RB1CC1/FIP200	Wild-type p53 inhibits autophagy by interacting with the human ortholog of yeast Atg17, RB1CC1/FIP200	(276)
Inhibited	PKR	Wild-type p53 inhibits autophagy by reducing double-stranded RNA accumulation and PKR (protein kinase RNA activation) activation	(277)

AICAR, 5-Aminoimidazole-4-carboxamide1-β-D-ribofuranoside; AMPK, Adenosine 5‘-monophosphate-activated protein kinas; ARF, Auxin response factor; Bcl-2, B-cell lymphoma-2; DAPK-1, Death associated protein kinase 1; HIF-1, Hypoxia-inducible factor 1 ; HSF-1, Heat shock factor 1; IGF-BP3, Insulin-like growth factor binding-protein-3; mTOR, Mammalian target of rapamycin; PI3K, Phosphoinositide 3-kinase; PKR, Protein kinase R; PTEN, Phosphatase and tensin homolog; RB1CC1, RB1-inducible coiled-coil 1; RPS6, Ribosomal protein S6; TIGAR, TP53 induced glycolysis regulatory phosphatase Gene; ULK1, Unc-51-like kinase 1; 4E-BP1, Recombinant human eukaryotic translation initiation factor 4E-binding protein 1.

p53 signaling plays a significant role in the development and progression of various lung diseases. Mutations in the p53 gene can disrupt its tumor-suppressor function, leading to uncontrolled cell growth, evasion of cell death, and genomic instability, all of which are hallmarks of cancer. Dysregulated p53 signaling has been linked to a poorer prognosis in lung cancer patients and resistance to certain anticancer therapies. Dysregulation of p53 contributes to abnormal repair processes in the lung tissue, leading to excessive collagen deposition, fibrosis, and impaired lung function. Moreover, activation of p53 can promote cell cycle arrest, DNA repair, or apoptosis, depending on the extent of damage while disruption of p53 function may impair the lung’s ability to repair and regenerate, exacerbating lung injury and contributing to disease progression.

## Function of autophagy

Autophagy serves as a mechanism for maintaining a stable pool of organelles by regenerating metabolic precursors and eliminating subcellular debris in response to diverse environmental stressors. In the presence of such stress, autophagy initiates cellular defense mechanisms by facilitating the removal of damaged organelles and ubiquitinated protein aggregates (90, 91). Under specific conditions of glucose or amino acid starvation, autophagy is compensatory to participate in the basic metabolic cycle of cells by acting on intracellular proteins, lipids, and other organic macromolecules (5). Specifically, autophagy plays a very important role in apoptosis, inflammation, and immunity. We will describe this in detail below.

## Autophagy in apoptosis

**Figure 4:**
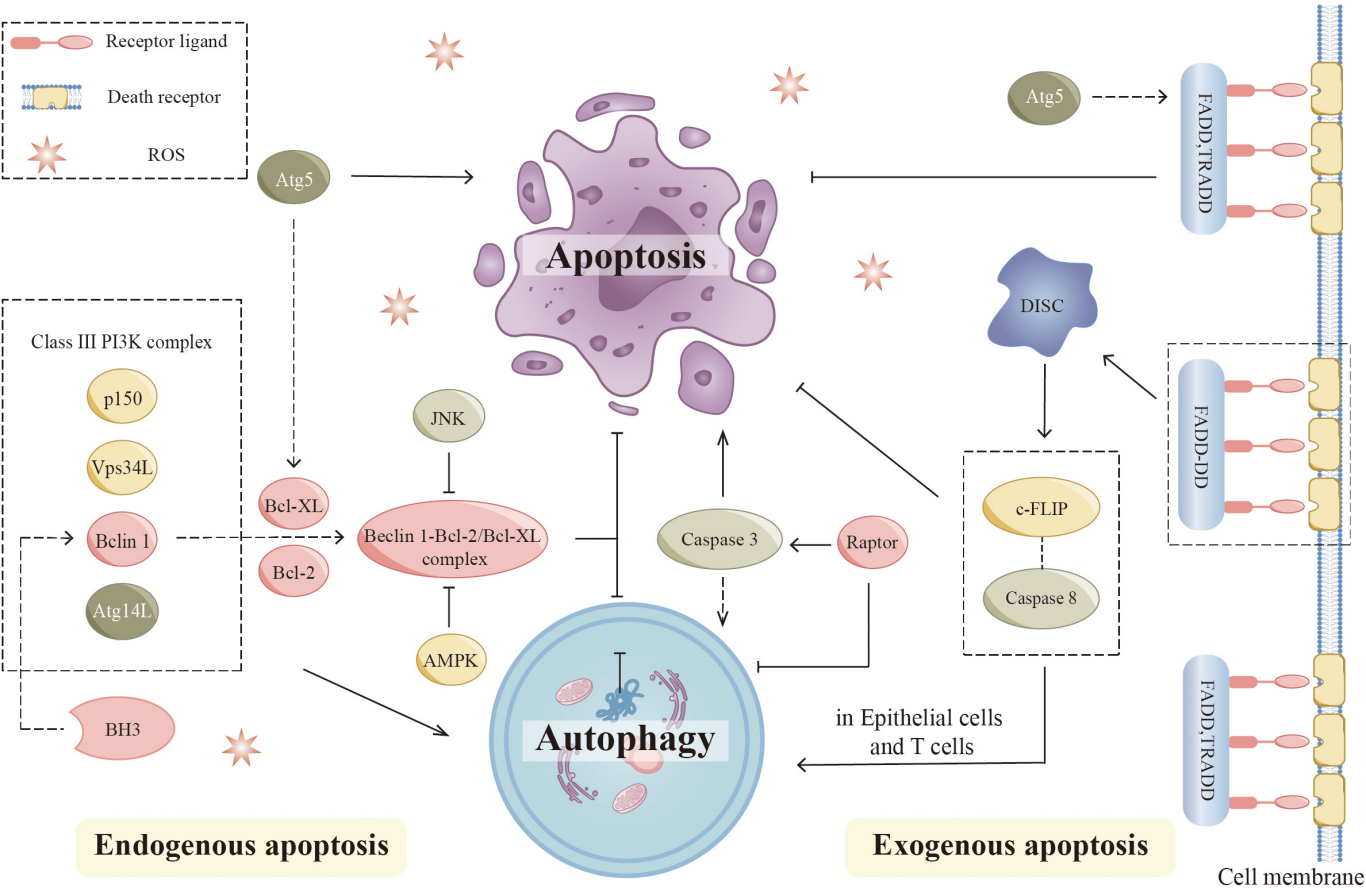
Autophagy and apoptosis. In endogenous apoptosis, the interaction of autophagic proteins with apoptotic proteins regulates this process. Bcl-2 family members, including Bcl-2 and Bcl-XL, can directly interact with Beclin 1 by binding to the BH3 domain. The JNK pathway promotes autophagy by preventing the association between Beclin 1 and Bcl-2 family proteins. AMPK also dissociates the Bcl-2-Beclin1 complex and promotes Beclin1-PI3K complex formation. Apoptosis signaling pathways may be affected by various autophagic proteins such as Atg5. Proteolytic fragments of Atg5 are able to promote apoptosis by inhibiting Bcl-XL. In extrinsic apoptosis, key components of DISC regulate autophagy during this process. Apoptosis and autophagy are affected by mutations in FADD, which create DD. The mutant (FADD-DD) was recruited to DISC in the absence of DED. By interacting with caspase 8 precursor and c-FLIP, this domain prevents the development of death receptor-induced apoptosis, while it can lead to excessive autophagy in epithelial cells and T cells. Atg5 can form a complex with FADD to affect the apoptosis process. AMPK, (Adenosine 5’-monophosphate-activated protein kinase); Bcl-2, (B-cell lymphoma-2); BH3, (Bcl-2 homolog3r); Caspase, (Cysteine protease); c-FLIP, (Cellular FADD-like IL-1β-converting enzyme-inhibitory protein); DD, (Death domain); DED, (Death effector domain); DISC, (Death-inducing signaling complex); JNK, (c-Jun-NH2-terminal kinase).

Cells can undergo apoptosis in response to intracellular signaling, extracellular signaling, and endoplasmic reticulum (ER) stress (**Figure 4**). Cysteine protease (caspase) induction and activation play a critical role in apoptosis. Endogenous apoptosis is also known as the mitochondrial pathway due to a mechanism closely related to the permeability of the mitochondrial membrane. This process is also strongly associated with the Bcl-2 protein family. Bcl-2, Bcl-XL, and Mcl-1 are negative regulators of apoptosis and protect cells from apoptosis when multiple types of cells are stimulated. Bax and Bak can undergo apoptosis by penetrating the mitochondrial membrane, releasing cytochrome c, and subsequently activating caspases. However, the exact mechanism by which these proteins promote apoptosis is unknown. Exogenous apoptosis requires the formation of a critical complex, the death-inducing signaling complex (DISC). Death receptors, including Fas, TNFR1, and TRAIL, are located on the cell surface and mediate apoptosis when activated. The production of DISC is initiated by the binding of death receptors to their corresponding ligands. When Fas binds to its ligand, activated Fas forms a DISC by binding connexin to the death domain (FADD). DISC then binds recruited pro-caspase 8 by interacting with another motif called the death effector domain (DED). Next, Pro-caspase 8 dimerizes and gains catalytic activity after degrading downstream substrates, producing and releasing heterotetrameric active caspase 8. Eventually, cells undergo extrinsic apoptosis. The ER stress pathway involves the buildup of incorrectly folded or unfolded proteins within the ER, which can arise from various factors such as infection, hypoxia, starvation, chemical influences, and deviations from homeostatic regulation of ER secretory functions. This accumulation leads to ER stress-induced apoptosis and triggers the unfolded protein response (UPR) pathway in response to the misfolding of proteins within the ER.

Recent research has indicated a strong connection between autophagy and apoptosis (92, 93). According to different experimental models, autophagy is associated with anti-apoptotic and pro-apoptotic effects (94). Several signaling mechanisms interact between apoptosis and autophagy. Autophagy proteins are involved in the regulation of apoptosis, while apoptotic proteins also influence the process of autophagy (95). Bcl-2 family members Bcl-2 and Bcl-XL, can directly interact with Beclin 1 by binding to the BH3 domain in the intrinsic apoptotic pathway (96, 97). Further studies showed that the anti-autophagic function of Bcl-2 mainly occurs in the ER and stabilizes Beclin-1 interaction with Bcl-2 through its 2Fe-2S cluster binding to Bcl-2. The c-Jun-NH2-terminal kinase (JNK) pathway is closely linked to apoptosis signaling. The JNK pathway can regulate the function of autophagy by affecting several key proteins (98). The JNK pathway promotes autophagy by preventing the association between Beclin 1 and Bcl-2 family proteins (99). In addition, AMPK can dissociate the Bcl-2-Beclin1 complex and promote the formation of the Beclin1-PI3K complex (100). Notably, mTOR is key in linking apoptosis and autophagy. It has been shown that loss of Raptor activates caspase 3, leading to mitochondrial abnormalities, which positively regulate apoptosis and autophagy (101).

Additionally, there is a complex link between the extrinsic apoptotic pathway and autophagy. Critical components of DISC regulate autophagy in this process. Excess autophagy occurs in fibroblasts, macrophages, and T cells when caspase 8 is inhibited or deficient (102, 103). DED is a protein interaction domain that can be found in pro-caspases and proteins in the apoptotic cascade that regulate caspase activation. Apoptosis and autophagy are also affected by FADD mutations, which produce abnormal death domains (DD). Mutants (FADD-DD) were recruited to DISC without DED. By interacting with pro-caspase 8 and cellular FADD-like IL-1β converting enzyme inhibitor protein (c-FLIP), this domain prevented the development of death receptor-induced apoptosis. Besides, it can cause excessive autophagy in epithelial cells and T cells (102, 104). Exogenous apoptotic signaling pathways can be affected by several autophagic proteins such as Atg5 (105). The knockdown of Atg5 exerts different effects on cell survival under different study conditions (106). Typically, proteolytic fragments of Atg5 can promote apoptosis by inhibiting Bcl-XL (107).

## Autophagy in inflammation and immunity

**Figure 5:**
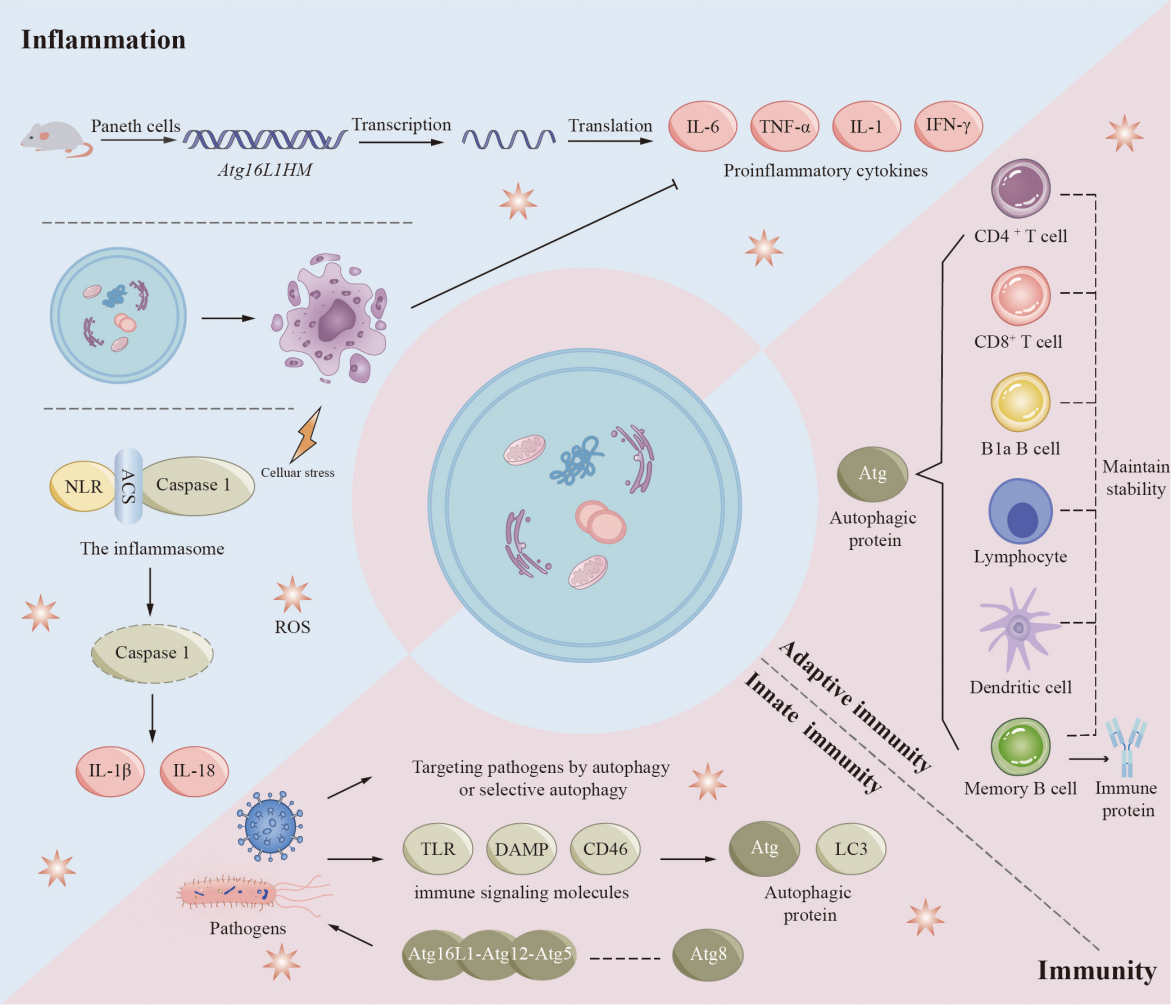
Autophagy in inflammation and immunity. Autophagy proteins play a role in inducing and suppressing immune and inflammatory responses, and immune and inflammatory signals play a role in inducing and inhibiting autophagy. Autophagic proteins play an important role in adaptive immunity, mainly including maintaining the normal number and function of immune-related cells such as B1a B cells, CD4^+^ T cells, and CD8^+^ T cells. Autophagy also plays a role in innate immunity when pathogens such as bacteria and viruses invade the human body. However, some pathogens are able to achieve their own survival by inhibiting, evading or even utilizing the autophagic process. Autophagy pathways and associated proteins also play crucial roles in regulating inflammatory responses. Increased transcription of pro-inflammatory cytokines and adipokines has been observed in mouse Paneth cells (Atg16L1HM), which contribute to the development of inflammation. Inflammasomes are important substances in the development of inflammation, and inflammasomes activated by various factors mediate the degradation and activation of caspase-1 and ultimately promote the synthesis and secretion of inflammatory factors (IL-1β and IL-18). Autophagy also removes cell debris generated by apoptosis, which in turn inhibits tissue inflammation.

The autophagy process influences immune and inflammatory responses in many diseases (**Figure 5**) (7). There is a complex interrelationship among autophagy, immunity, and inflammation. Autophagic proteins play a role in inducing and suppressing immune and inflammatory responses. Similarly, immune and inflammatory signals play a role in inducing and inhibiting autophagy. Autophagy provides new insights into the prevention and treatment of infectious, autoimmune, and inflammatory diseases by balancing the benefits and drawbacks of immune and inflammatory responses.

Autophagy regulates various immune responses during infection. In many experiments, we have found that mutations in autophagy genes increase susceptibility to certain diseases (108–112). Studies performed on human genetics have revealed important clues regarding xenophagy, autophagic proteins that affect pathogen replication or survival, and the general immune system. Numerous studies have demonstrated the significance of autophagy in the human cellular defense mechanisms against mycobacterial infections (113). Autophagy genes play a significant role in regulating host genes for Mycobacterium tuberculosis (Mtb) replication (114). Autophagy may be a crucial component of TB drug resistance. At the same time, to survive *in vivo*, some viruses and bacteria have evolved different methods of adaptation to autophagy. They can prevent the occurrence of autophagy by inhibiting the foremost steps of autophagy or/and the production of autophagosomes, avoid protein modification or interfere with the recognition of autophagy by autophagy signaling, and even promote self-replication and survival using autophagy-related proteins. For example, human immunodeficiency virus (HIV), Kaposi’s sarcoma-associated herpes virus inhibits antiviral capacity and immune properties *in vivo* by affecting key pathways of autophagy. HIV envelope proteins activate mTOR signaling and prevent HIV transfer to CD4 + T cells. Kaposi’s sarcoma-associated herpesvirus prevents LC3-II production by interacting with Atg3 (115). Bacteria also have multiple strategies to avoid degradation. By disguising themselves, several bacteria can evade autophagic recognition in the cytoplasm. For example, VirG is a protein present on the bacterial surface and is necessary for Shigella to be targeted by autophagosomes. Atg5 can prevent its interaction with VirG by competitively binding to IcsB, an effector of Shigella (116). Several cytoskeletal proteins of cells are ActA-dependent (117). This feature allows bacteria to masquerade as their host organelle (117). Listeria protein ActA interacts with the intracytoplasmic actin polymerization machinery, thereby blocking binding to ubiquitin, recruitment of p62, and autophagy targeting (117). Several pathogens are also able to benefit themselves using components of autophagy in membrane trafficking, including poliovirus, rotavirus, coronavirus, dengue virus, and hepatitis B and C viruses (113, 118).

Autophagy is also regulated by immune signaling molecules, including innate and adaptive immunity. Although the regulatory mechanism of autophagy by most immune-related signaling molecules is currently unknown, some findings provide clues. NOD1 and NOD2, two typical NLRs (NOD-like receptor cryopyrin protein), can be activated by specific components of bacterial peptidoglycan. In response to bacterial infection, activated NOD1 and NOD2 interact with ATG16L1 to induce autophagy (119). NOD2 mutations associated with Crohn’s disease have been found to influence ATG16L1 recruitment and bacterial co-localization with LC3 (119). Presumably, in innate immunity, the ATG5-ATG12-ATG16L1 complex interacts with members of the ATG8 family and may stimulate pathogen-induced autophagy or enhance the ability of selective autophagy to target pathogens (120). Various cytokines, including but not limited to CLCF1, LIF, IGF1, FGF2, and the chemokine SDF1 (also called CXCL12) may have a broader role in controlling autophagy (121). Autophagy also plays a crucial role in adaptive immunity. Multiple regulatory pathways of autophagy possess the capacity to affect both the functionality and stability of the immune system, in addition to influencing antigen presentation. B1a B cells, CD4 + T cells, CD8 + T cells, and fetal hematopoietic stem cells rely on autophagic proteins to maintain their numbers (122–124). Thymic clearance of autoreactive T cells is an important function of autophagy in immune system development and homeostasis (123). Epithelial cells of the thymus are highly autophagic. Mutations in Atg5 in thymic epithelial cells result in altered autoimmunity and specific immunity of certain MHC class II-restricted T cells (125). In addition, autophagy may play an important role in the differentiation of lymphocytes by indirectly affecting the expression of cytokines. During antigen presentation, autophagy proteins present endogenous antigen MHC class II to CD4 + T cells, enhance cross-presentation of antigen-providing cells with CD8 + T cells, and facilitate cross-presentation of phagocytosed antigens by dendritic cells to CD4 + T cells (126–128). Autophagy also contributes to memory B cell maintenance and regulates immunoglobulin secretion (129–131).

Recent findings have shown that autophagy is closely associated with the development of certain chronic inflammatory diseases, such as Crohn’s disease, systemic lupus erythematosus (SLE), and other autoimmune diseases (132, 133). In animal models and human diseases, autophagic failure is usually characterized by dysregulation of inflammation (134). Its main role is to regulate inflammatory transcriptional responses. Increased transcription of proinflammatory cytokines and adipokines has been observed in Paneth cells (Atg16L1HM) of Atg16L1 subtype mice (119, 135–137). Inflammasomes are another important target of autophagic proteins in inflammatory signaling. Inflammasomes are multiprotein complexes containing NLR, adaptor protein ASC, and caspase 1. Inflammasomes can be stimulated by infection or other stress-related pathways. Activated inflammasomes mediate the degradation and activation of caspase-1 and ultimately promote synthesis and secretion of IL-1β and IL-18 (138, 139). In addition, activation of the NALP3 inflammasome is increased in Beclin 1 and LC3B gene-deficient monocytes (140, 141). This enhancement ultimately facilitates the activation of IL-1β and IL-18. The autophagic process can also suppress tissue inflammation by removing apoptotic corpses. During developmentally programmed cell death, autophagy induces xenophagic clearance in dying apoptotic cells by generating ATP-dependent phagocytic signals (142). Increasing evidence suggests that autophagic proteins are required for TLRs mediated phagolysosomal pathways (142). To clear inflammatory sources such as exogenous inflammatory sources (e.g., bacterial viruses) and endogenous pro-inflammatory sources (e.g., damaged organelles, aggregates), autophagic cargoes are usually regulated by ubiquitin and are regulated by a type called SLR (sequestrate-like receptor: p62 [SQSTM1], NBR1, OPTN, NDP52, TAX1BP1, etc.) (143).

## Methods for measuring autophagic activity

Currently, the most effective methods for analyzing autophagy *in vitro* and *in vivo* remain significantly controversial, due to the complexity of the autophagic process. The measurement of autophagic activity can be divided into two categories: counting autophagosomes and measuring autophagic flux.

Currently, three primary methodologies are employed to assess the number of autophagosomes: electron microscopy (144), Western blot (WB) analysis (145), and fluorescent protein labeling techniques (146). Electron microscopy observation of autophagic structures is the most traditional method. Morphological alterations occurring at various stages of autophagy can be directly visualized using a transmission electron microscope, allowing for an initial assessment of the autophagic phase. Electron microscopy showed damaged organelles in cells undergoing autophagy. In the case of mitochondria, vacuolated bilayer membrane-like structures, or vacuolated structures of bilayer membranes, i.e., autophagosomes, can be observed around them (146, 147). LC3 runs through the whole autophagic process and is currently recognized as an autophagic marker. Changes in the LC3-II/I ratio can be detected using WB to assess the intensity of autophagy. In addition, autophagy can be detected using the property of green fluorescent protein (GFP) quenching in acidic environments (146). Based on GFP-LC3, the researchers developed the GFP-RFP-LC3 tool, a method that allows observation of autophagy in individual cells. Keima is a unique fluorescent protein that is independent of LC3 and suitable for monitoring nonselective autophagy and microautophagy (148). Keima can additionally serve as a tool for the detection of organelle autophagy when conjugated with organelle-specific markers. Scholarly investigations have indicated that an increased presence of autophagosomes or LC3B-II within the system correlates with enhanced proteolytic activity. However, there is no clear correlation between autophagy activity and the abundance of autophagosomes or LC3B proteins (24, 146, 149). For this reason, dynamic measurements of autophagic flux are required (146).

A prominent contemporary approach for assessing autophagic flux involves the observation of LC3 turnover. This approach relies on LC3B-II pooling at autophagosome membranes. When cells were treated with lysosomal reagents (e.g., ammonium chloride) or lysosomal protease inhibitors (e.g., chloroquine), the degradation of LC3-II was blocked, resulting in the accumulation of LC3-II. Thus, the difference in LC3-II amounts between samples represents the amount of LC3 that is delivered to lysosomes for degradation (150). Second, the amount of total cellular LC3 can be quantified by immunoblot analysis or flow cytometry or qualitatively observed by fluorescence microscopy, which is inversely proportional to autophagic flux. In addition to LC3, several groups have developed some specific macrophage substrates to monitor autophagic flux, such as p62/SQSTM1 (151, 152), BRCA1 gene 1 protein (NBR1) (151), betaine-homocysteine s-methyltransferase (153), and polyglutamine protein aggregates (154).

## Autophagy in lung diseases

### COPD

**Figure 6:**
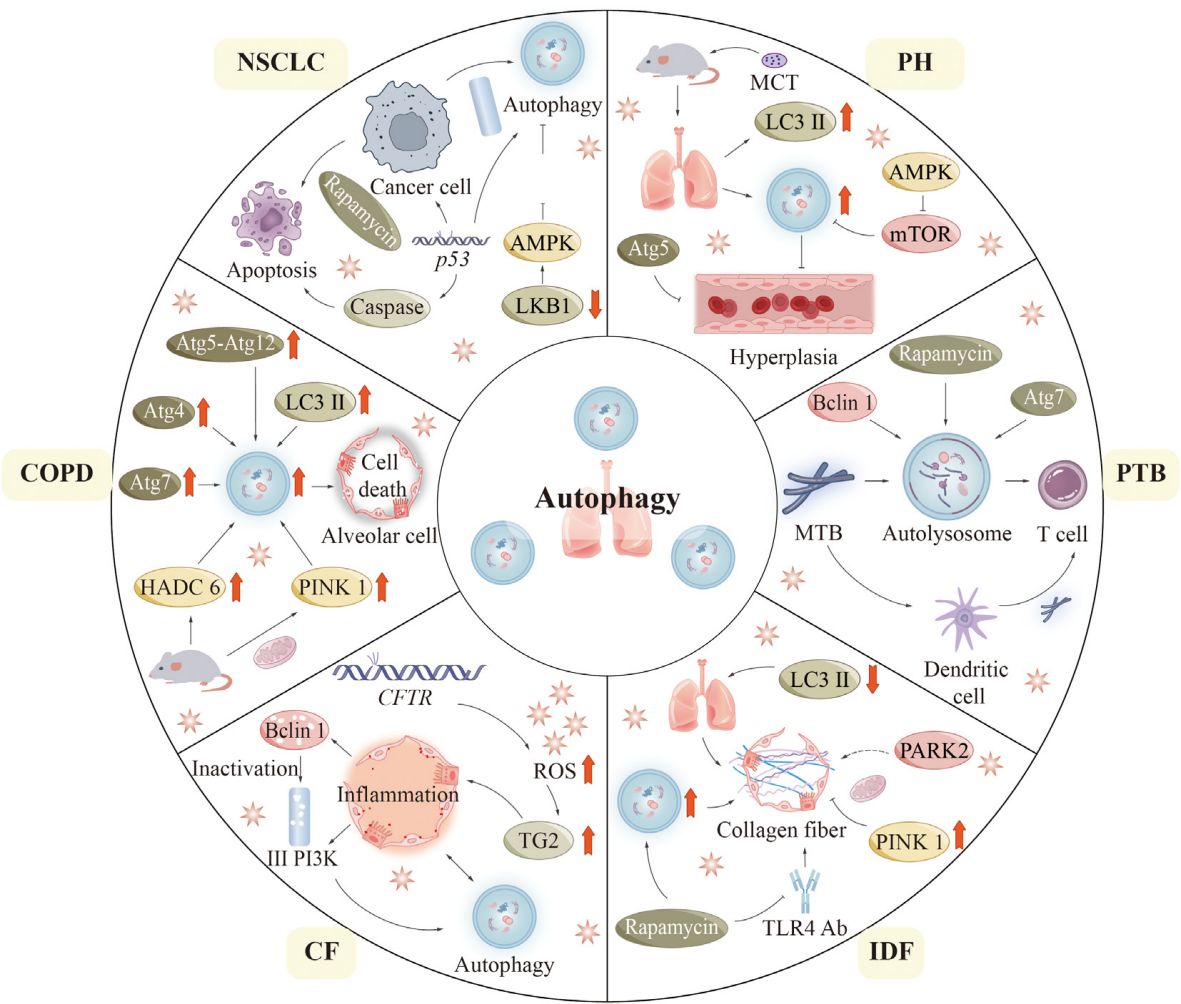
Autophagy in lung diseases. In this figure, we summarize the pathogenesis related to the process of autophagy in six pulmonary diseases: COPD, CF, IPF, PTB, PH, and NSCLC. CF, (Cystic fibrosis); COPD, (Chronic obstructive pulmonary disease); IPF, (Idiopathic pulmonary fibrosis); NSCLC, (Non-small cell lung cancer); PH, (Pulmonary hypertension); PTB, (Pulmonary tuberculosis).

COPD is a chronic inflammatory pulmonary disease connected with smoking, which is the third most common death factor around the world and consists of 3 primary disease states: chronic bronchitis or proximal airway mucus hypersecretion; emphysema or peripheral lung destruction and loss of alveolar attachments; and small airway disease characterized by inflammation and airway remodeling (**Figure 6**) (155, 156).

In 2000, autophagic vacuoles were detected in liver specimens lacking alpha-1 antitrypsin, indicating the potential role of autophagy in lung disease (157). COPD pathogenesis is not fully understood but may be associated with aberrant cellular responses of bronchial cells and lung cells to CS (cigarette smoke) (158, 159). In the setting of COPD, autophagy-promoting epithelial cell death was shown to be a potential mechanism (160, 161). As compared to healthy individuals, COPD patients have increased levels of LC3B-II and autophagy-related proteins including ATG4, ATG5-ATG12, and ATG7 (160). In addition, it was observed under electron microscopy that the formation of autophagosomes was also markedly increased in lung tissues from COPD patients compared with control lung tissues (160). Mice exposed to CS are usually used as an experimental model of COPD. In lung tissue, mice subjected to cigarette smoke exposure exhibit elevated levels of autophagic proteins and an increased presence of autophagosomes. Interestingly, mice deficient in LC3B and autophagy proteins are resistant to CS-induced pathological changes (161, 162). These findings indicate that the autophagy pathway may contribute to the progress toward COPD in some specific circumstances (160, 161).

Histone deacetylase 6 (HDAC6) is a critical regulator of primary ciliary uptake. Studies have shown that HDAC6 is involved in the degradation of autophagy in cells (162). Shortened cilia and increased HDAC6 are observed in respiratory epithelial cells treated with CS exposure. Cilia shortening induced by CS is inhibited in mice that lack autophagic protein and HDAC6 (162). This result reflects the importance of pathological changes of HDAC6 in respiratory epithelial cells. Consequently, autophagy plays a role in the HDAC6-mediated degradation of cilia within airway epithelial cells in experimental models of COPD (163). Some studies have reported the involvement of mitochondrial in (164, 165). A key mitophagy protein, phosphatase and angiotensin homolog (PTEN) -induced putative kinase 1 (PINK1), has been found to be increased in the lungs of COPD patients (166). Genetic defects in PINK1 and inhibition of mitophagy with drugs showed resistance to COPD pathology in CS-exposed mice (166).

### Cystic fibrosis

Cystic fibrosis (CF) is an autosomal recessive disorder due to mutations in the CF gene located in the 7th pair of chromosomes, which can cause serious damage to the lungs, digestive system, and other organs of the body (167–169). Mutations in related CF genes affect the expression of cystic fibrosis transmembrane conductance regulators (CFTR). It is most typical to have a 508-phenylalanine deletion in the CFTR gene (CFTRF^508del^) (168). The primary characteristic of cystic fibrosis (CF) within the respiratory system is the overproduction and subsequent accumulation of mucus in the airways. This pathological change can secondarily cause recurrent bronchial infections and airway obstruction. In epithelial cells, mutations in CFTR lead to increased ROS formation. Accumulated ROS promotes tissue transglutaminase 2 (TG2) production. Excessive TG2 is an important cause of inflammatory reactions in CF (170, 171). These complex responses lead to the loss of Beclin 1 and class III PI3K complex function, further affecting autophagic function. Notably, the enhancement of autophagy through the overexpression of Beclin 1 has been shown to enhance inflammatory responses (170), indicating that the autophagic system is essential for the clearance of protein aggregates. In a similar vein, mice with the F508del-CFTR mutation demonstrate reduced levels of Beclin 1 expression (172). Significant risk of morbidity and mortality exists in CF patients due to pseudomonas aeruginosa infection. Experimentally, defective autophagic function resulting from reduced levels or loss of function of BECN1 renders mice lungs more vulnerable to pseudomonas aeruginosa infection (173).

### Idiopathic pulmonary fibrosis

Idiopathic pulmonary fibrosis (IPF) is the most common form of idiopathic interstitial pneumonia in clinical practice and is a chronic pulmonary disease of unrecognized etiology (174, 175). Although IPF has epithelial origins, it displays abnormal adaptive immune responses, such as T-cell and B-cell dysregulation (176). The levels of LC3-II expression in lung tissue from patients diagnosed with IPF are significantly reduced compared to those observed in healthy individuals (160, 177). Autophagy seems to play a protective role in the development of IPF. In experimental models of IPF, rapamycin inhibits lung fibroblasts’ expression of fibronectin and alpha-smooth muscle actin through its up-regulation of autophagy (178). In addition, the pro-autophagic effect of rapamycin is shown to promote collagen formation in lung epithelial cells (177). Rapamycin also inhibits pulmonary fibrosis induced by Toll-like receptor 4 (TLR4) antibodies or bleomycin in mice (177–179). Furthermore, in the absence of autophagy genes or when autophagy is suppressed pharmacologically, transforming growth factor (TGF) activates lung fibroblasts (177, 178).

In IPF, researchers found clusters of malformed mitochondria in lung epithelial cells, particularly in alveolar type II cells (AECIIs) (180). In addition, microarray analysis showed decreased PINK1 in lung tissues from IPF patients (180). The knock-down PINK1 mice displayed increased mitochondrial depolarization and expression of pro-fibrotic factors (180, 181). The mechanism of the antifibrotic effect of PINK1 in lung epithelial cells is reflected in the prevention of cell death by preserving the morphology and function of mitochondria (180, 181). It has been suggested that PARK2, an important mitophagy-related molecule may be linked to the pathogenesis of IPF (182). Mitophagy is activated in alveolar macrophages from IPF patients and mice treated with bleomycin. Whereas increased apoptosis of macrophages is found in mitophagy-deficient mice, which prevents them from pulmonary fibrosis.

### Pulmonary hypertension

Pulmonary hypertension (PH) is a disease of abnormally high blood pressure in the pulmonary arteries. PH is predominantly defined by the remodeling of pulmonary vasculature, a multifaceted and progressive phenomenon that ultimately results in right heart failure and mortality. Studies have investigated how autophagy acts in PAH pathogenesis, but conclusions remain disputed. Experimental mice with chronic hypoxia showed increased levels of LC3B and autophagosomes in their lungs (183). Furthermore, a higher prevalence of autophagic vacuoles was noted in lung tissue subjected to hypoxia. Mice deficient in MAP1LC3B (MAP1LC3B −/−) that were exposed to chronic hypoxic conditions demonstrated more pronounced PH values in comparison to their wild-type counterparts. PH values included right ventricular systolic pressure and vessel wall thickness. An elevation in angiogenesis within pulmonary artery endothelial cells has been noted in cases of persistent PH in Beclin 1-null mice (184). ATG5-targeting siRNA has been found to directly disrupt autophagy to inhibit the proliferation process of rat pulmonary artery smooth muscle cells. The AMPK signaling pathway, recognized as a crucial component of autophagy, significantly results in the process of autophagy in cardiomyocytes. Research has shown that pharmacological inhibition of the AMPK pathway increases cardiomyocyte mortality, suggesting a protective effect on AMPK-associated cardiomyocyte autophagy. Monocrotaline (MCT) is a commonly used drug in animal experimental models of induced PH. Recent research indicates that in rats treated with monocrotaline (MCT), the expression of phospho-mTOR in the right ventricle is down-regulated, while the expression of phospho-AMPK is elevated at the 2 and 4-week marks. Conversely, at the 6-week interval, there is an up-regulation of phospho-mTOR expression and a decrease in phospho-AMPK expression in the right ventricle of MCT-treated rats (185). This suggests that the AMPK-mTOR autophagy signaling pathway is involved in regulating autophagy in pulmonary hypertension rats. It has already been demonstrated that rapamycin treatment can prevent right ventricular hypertrophy and dysfunction through activation of the autophagy pathway in animal models. The findings indicate that autophagy could potentially be a contributing factor to human vascular disease (186). However, these findings are derived from static measurements. Additional experimental investigations are necessary to elucidate the relationship between human vascular disease and autophagy (183).

### Pulmonary tuberculosis

PTB is a chronic and long-term pulmonary disease caused by Mtb infecting the human lung, and it is the predominant manifestation of tuberculosis. Mtb is classified as an intracellular pathogen that releases a diverse array of effector proteins within host cells. These proteins subsequently disrupt cellular signaling pathways, thereby influencing normal cellular functions. It ultimately promotes its survival in host cells and leads to host cell pathology. During the initial stages of infection, innate immune responses are stimulated, and inflammatory cells are recruited to the lungs. Mtb evades and eliminates innate immune cells, spreads to the draining lymph nodes, and triggers a specific T-cell response that promotes the formation of granulomas at the sites of pulmonary infection (187). Inflammatory granulomas are believed to lead to lung tissue damage, and pulmonary fibrosis, and progressively develop chronic and persistent clinical manifestations of PTB (188).

Mtb inhibits phagosome-lysosome fusion, allowing it to persist in the phagosome during maturation. Autophagy is important for the elimination of Mtb. *In vitro*, rapamycin or starvation-induced autophagy promotes the conversion of Mtb phagosomes into autolysosomes, which contain more antimicrobial chambers (e.g., antimicrobial peptides) than conventional phagosomes (129, 189). Macrophages are also enhanced in their ability to present mycobacterial antigens by autophagy (197). Moreover, phagolysosomal fusion is found to be inhibited when cells are infected with Mtb in macrophages lacking Beclin 1 and ATG7 (189). This result could preliminarily prove that autophagy is advantageous for killing tubercle bacilli. However, the specific mechanism of the defense effect of autophagy proteins on Mtb in humans is unknown. In addition, recent *in vitro* studies have demonstrated increased Mtb replication in HIV-infected macrophages co-infected with Mtb when autophagy is activated by the suppression of the mTOR pathway (190). A recent study found that certain autophagic mechanisms acting on phagocytes are critical mechanisms to target Mtb, known as xenophagy. The embryonic exogenic homeobox 1 (ESX-1) secretion system is a virulence factor of Mtb (191). ESX-1 causes Mtb DNA exposure to the host cytoplasm through phagosome permeation (192). DNA exposed to the cytoplasm is detected and ubiquitinated by cytoplasmic DNA sensor molecules (e.g., STING) (192). Ubiquitinated DNA attaches to LC3 via several proteins like p62 and nucleoporin 52. Consequently, it is encapsulated in autophagosomes to fuse with lysosomes (192).

### Non-small cell lung cancer

The mechanism of action of autophagy in cancer has repeatedly been described as a double-edged sword. The role that autophagy-related cellular pathways play in the pathological progression of NSCLC is being extensively investigated. Mutations in genes involved in the mTOR signaling pathway may be associated with malignant proliferation of cells. Mutations in genes in the mTOR pathway, such as KRAS, epidermal growth factor receptor (EGFR), LKB1, PTEN, PIK3CA, AKT1, EGFR, PIK3CA, and PTEN, have some relationship with the development of NSCLC (29). Research indicates that the anticancer efficacy of LKB1 is diminished in NSCLC. The researchers propose that this reduction may facilitate tumor proliferation via the LKB1-AMPK-mTOR signaling pathway (193). Rapamycin causes endogenous apoptosis of cancerous cells, which in turn inhibits tumor growth in mouse models of NSCLC (194, 195). *In vitro* lung cancer models, rapamycin enhances apoptosis and autophagy (196). The PI3K signaling pathway serves as a primary regulator of autophagy, with its activation leading to the inhibition of autophagic processes in cancerous cells. Furthermore, the activation of this pathway enhances the production of tumor-promoting antigens, thereby facilitating the process of carcinogenesis. Mutations in p53 are also important correlates of tumorigenesis. p53 is one of the most frequently mutated genes and is present in 45% – 70% of adenocarcinomas and 60% – 80% of squamous cell carcinomas (196). The pathogenesis of NSCLC depends heavily on the absence of the p53 gene (197). p53 can be present in the cytoplasm and nucleus. Under cellular stress, p53 can translocate into the nucleus (197). The p53 protein, located in the nucleus, experiences conformational alterations that enable it to function as a transcription factor. This activity facilitates the upregulation of numerous pro-apoptotic proteins, thereby making sense in the regulation of both endogenous and exogenous apoptotic pathways (197). p53 can also translocate to the mitochondrial surface and directly bind to Bcl-2 family proteins to promote endogenous apoptosis (197, 198). In addition to this, p53 promotes the expression of Apaf-1 and caspase-6 and promotes extrinsic apoptosis (198). p53 also plays a role in regulating the autophagic process. An autophagy-induced response was observed in mice whose p53 expression was blocked using cypermethrin-α. And p53-null cells also showed enhanced autophagy compared with wild-type cells. In addition, cytoplasmic p53 can interact with FIP200, which in turn competitively inactivates autophagy.

### Other lung diseases

In addition to the above lung diseases, autophagy is also greatly related to the occurrence and development of many other lung diseases, such as asthma, COVID-19, and atypical pneumonia.

Autophagy plays a complex role in the pathophysiology of asthma and may be deleterious or beneficial. Autophagy can affect inflammatory response, airway remodeling, immune regulation, and other aspects, and is an important field of asthma treatment research. Polymorphisms in the autophagy-related gene Atg5 are strongly associated with asthma (199). In respiratory epithelial cells of patients with severe asthma, the expression level of Atg5 protein is increased, and this phenomenon is closely related to the deepening of the degree of fibrosis in the lower cell layer as well as the increase of collagen-1 expression (200). IL-13 plays a crucial role in the development of T2 asthma (201). *In vitro*, IL-13 stimulated goblet cell production and secretion of MUC5AC protein from human respiratory epithelial cells (202). This process is associated with activation of the autophagic process, which is blocked when expression of Atg5 is inhibited. In addition, inhibition of autophagy also affects IL-13 production in response to ROS (203). In addition, in asthmatic patients, airway epithelial cells initiate autophagy by inhibiting mTORC1 signaling in response to IL-13 or IL-33 (204). Bronchial fibroblasts showed enhanced mitophagy accompanied by increased expression levels of PINK1 and Parkin protein in severe asthma, which may be an adaptive response against mitochondrial dysfunction in asthmatic cells (205).

COVID-19 is caused by a coronavirus called SARS-CoV-2, and prior research has indicated that autophagy may play a dual role in the context of coronavirus infection (206). Autophagy can degrade coronavirus, enhance inflammatory responses, and modulate inflammation in neutrophils (207). At the same time, it also promotes antigen presentation and provokes immunity against coronavirus (208). However, double-membrane vesicles of autophagosomes facilitate the sequestration of the virus from external immune responses, thereby serving as sites for viral replication and transcription (207). In addition, nonstructural protein 6 (NSP6) of novel coronavirus assists the virus in escaping host innate immunity by activating autophagy. Recent studies have shown that overexpression of SARS-CoV-2 papain-like protease cleaves ULK1 and disrupts the formation of ULK1-ATG13 complex to block intact host autophagy (209). Corona virus also inhibits BECN1 and activates autophagy inhibitors (SKP2 and AKT1) to prevent autophagosome fusion with lysosomes to limit autophagy signal transduction (210, 211). Interestingly, compared with classical SARS-CoV, ORF3a of SARS-CoV-2 can separate homotypic fusion and protein classification components, thereby inhibiting fusion of autophagosomes and lysosomes, which is a unique feature of SARS-CoV-2 inhibition of autophagy (212, 213).

Autophagy is also important in atypical pneumonia, for example, infections caused by Chlamydia pneumonia(CP), Mycoplasma pneumonia, and Legionella. In CP, it has been shown to limit intracellular CP growth *in vitro* by inhibiting autophagy, but *in vivo*, research has demonstrated that the impairment of autophagy in myeloid cells is associated with increased mortality, potentially resulting from intricate antagonistic interactions between inflammasomes and autophagy (214). Post-infection with Mycoplasma pneumonia, the activation of autophagy may correlate with the severity of the disease, and both excessive activation and suppression of autophagy could influence the progression of the illness. Membrane lipids of Mycoplasma pneumonia can activate autophagy through TLR4 and promote the production of inflammatory factors such as TNF-α and IL-1β, exacerbating the inflammatory response (215). Legionella can inject effector proteins into host cells via its type IV secretion system (Dot/Icm) to avoid autophagy and survive. Nevertheless, the autophagy gene Atg7 can also exert its effect by assisting macrophages to clear bacteria (216).

## Autophagy as a potential therapeutic target for the treatment of lung diseases

In the human body, autophagy is essential to maintain the normal functioning of tissues and organs as well as the development of diseases. Thus, targeting autophagy may be useful in the treatment of disease, but may also exacerbate disease deterioration. Because the autophagic process can help clear harmful protein aggregates and damaged organelles. However, excessive autophagy or dysregulation of autophagy may be harmful to cells. In lung diseases, the role of autophagy is particularly complex. On the one hand, it can clear pathogens and damaged cells in the lungs and help resist infection and inflammation. On the other hand, if the autophagic process is dysregulated, it may lead to damage and dysfunction of lung tissue. Therefore, using autophagy as a target for the treatment of lung diseases requires great caution. If the treatment strategy is not appropriate, it may exacerbate the condition rather than improve it. How to balance the activation and inhibition of autophagy to achieve the best therapeutic effect is currently a major difficulty in autophagy-targeted therapy (**Table 2**).

**Table 2:** The mechanism of autophagy-related targets in lung diseases.

Lung diseases	Drug	Autophagy-related targets	Study subject	Mechanism	Reference
COPD	Cysteamine	ROS, Beclin-1, p62	Beas2b cells, C57BL/6 mice, and human (GOLD 0-IV) lung tissues	Cysteamine-induced autophagy can reduce aggregate formation, CS-induced alveolar senescence/death, and emphysema progression.	(278)
	Parkin activators (Preclinical)	PRKN	HBEC	PRKN levels attenuate COPD progression by modulating PINK1-PRKN-mediated mitophagy.	(279)
	NaHS, PAG	COX2	Human lung cells, Bronchial epithelial BEAS-2B cells	H2S inhibits the iron autophagy pathway mediated by NCOA4 and inhibits the ferritin response mediated by lipid peroxidation by activating Nrf2 and PPAR-γ signaling pathways.	(280)
	U75302	LTB4, TFEB	HBE	Inhibition of the LTB4/BLT1 pathway by U75302 promotes autolysosome formation and degradation in response to CS exposure.	(281)
	Gemfibrozil/Laccatin	TFEB	Beas2b cells and C57BL/6 mice	Gemfibrozil/laccein can control CS-induced airway inflammation and autophagic injury by up-regulating TFEB.	(282)
	Ghrelin	NF-κB, AP-1	HBEC	Ghrelin inhibits excessive inflammatory pathways and autophagy induced by particulate matter and/or cigarette extracts in bronchial epithelial cells.	(283)
	Klotho	AKT, ERK	MH-S cells	Klotho inhibits CSE-induced autophagy by down-regulating IGF-1R, Akt and ERK phosphorylation in alveolar macrophages.	(284)
	PFI-103	FSTL1	Human lung cells, C57BL/6 mice	FSTL1 can activate PI3K/Akt signaling pathway and AMPK signaling pathway to regulate autophagy.	(285)
	HMGB1	NF-κB	Human lung cells, C57BL/6 mice	HMGB1 abrogates migration and NF-κB activation in CSE-treated lung macrophages by inhibiting autophagy.	(286)
	Puerarin	FUNDC1	HBEC	Puerarin inhibits FUNDC1-mediated mitochondrial autophagy and CSE-induced apoptosis of human bronchial epithelial cells by activating the PI3K/AKT/mTOR signaling pathway.	(287)
	MitoQ	ROS	HUVEC	MitoQ maintains mitochondrial function by reducing ROS production and excessive autophagy.	(288)
	Vardenafil	AMPK, mTOR	16 HBE cells, C57BL/6 mice	Vardenafil activates autophagy via the AMPK/mTOR signalling pathway	(289)
	Sodium tanshinone IIA sulfonate	PI3K, mTOR	ARPE-19 cells	Sodium tanshinone IIA sulfonate can increase PI3K and mTOR transcription, activate the PI3K/AKT/mTOR pathway, and reduce autophagy.	(290)
	Silymarin	ERK, p38	Beas-2B cells	Silymarin can attenuate inflammatory responses by intervening in crosstalk between autophagy and the ERK MAPK pathway	(291)
	Tiotropium, Olodaterol	JNK	BEAS-2B cells	Tiotropium/olodaterol protects bronchial epithelial cells from CSE-induced injury by inhibiting activation of autophagy and upregulation of JNK phosphorylation.	(292)
	Taurine	NADH	HBEC	Taurine triggers autophagy in C/EBPα/mitochondria/ATG7 pathway.	(293)
	Resveratrol	Notch1	HUVEC	RESV protects against CSE-induced apoptosis by regulating Notch1-mediated autophagy priming.	(294)
	Epoxyeicosatrienoic acids	PI3K/Akt, mTOR, p62	BEAS-2B cells	EETs inhibit autophagy via PI3K/Akt/mTOR signaling	(295)
	Quercetogetin	PINK1, DRP-1	BEAS-2B cells	QUE inhibits CSE-induced mitochondrial dysfunction and mitophagy by inhibiting phosphorylated (p) -DRP-1 and PINK1 expression.	(296)
CF	Azithromycin	A53T α-synuclein, Q74 huntingtin protein	HeLa, COS-7 cells	Azithromycin blocks autophagic clearance and autophagosome acidification,	(297)
	Myriocin	FA, SREBPF1	IB3-1 cells	Myriocin attenuates inflammation and activates TFEB-induced stress responses, enhances fatty acid oxidation and promotes autophagy.	(298)
	AR-13	CFTR, LC3-II	Human heparinized blood samples	AR-13 treatment increases CFTR and LC3-II expression in epithelial cells.	(299)
	Fatty Acid Cysteamine Conjugates	CFTR	HBEC	It enhances correction of misfolded F508del cystic fibrosis transmembrane conductance regulator (CFTR) by activating autophagy.	(300)
	cysteamine plus epigallocatechin gallate	CFTR	Human nasal epithelial cells	cysteamine plus epigallocatechin gallate rescues CFTR function when autophagy is active and improves CFTR function by expressing proteins that are rescuable	(301)
	Givinostat	CFTR	R77C fibroblasts	Givinostat synergistically improves trafficking efficiency by inducing the F508del CFTR mutant.	(302)
	Cysteamine	CFTR	CF mice	Cysteamine can rescue the function of the F508del-CFTR mutant, thereby restoring bacterial internalization and clearance through processes involving upregulation of the pro-autophagic protein Beclin 1 and reconstitution of the autophagic pathway.	(303)
IPF	Spermidine	mTOR, PI3K/AKT, ATG7	Primary human lung fibroblasts, C57BL/6 mice	Spermidine modulated autophagy by activating the expression of key autophagic molecules such as LC3-II, beclin-1, and ATG7 as well as inhibiting mTOR, a downstream mediator of PI3K/AKT signaling.	(304)
	Eupatilin	Sestrin2, PI3K/Akt, mTOR	MRC-5 cells	Eupatilin ameliorates lung fibrosis by activating the Sestrin2/PI3K/Akt/mTOR-dependent autophagy pathway.	(305)
	Fu-Zheng-Tong-Luo formula	Janus kinase 2, transcription 3	Growth factor-β-induced lung fibroblast model	Fu-zheng-Tong-luo formula promotes autophagy by controlling Janus kinase 2/signal transducer and activator of transcription 3 pathway.	(306)
	Niclosamide Ethanolamine Salt	PI3K, mTOR, Beclin-1	A549 cells, C57BL-6J mice	NEN inhibits PI3K-mTORC1 downstream signaling and Beclin-1 independent autophagy, contributing to inhibition of TGF-β1-induced EMT and collagen deposition in epithelial cells and primary human fibroblasts	(307)
	Fibroblast growth factor 21	PI3K/AKT, mTOR	C57BL/6 mice	FGF21 ameliorates IPF by inhibiting PI3K-AKT-mTOR signaling pathway and activating autophagy	(308)
	Astragaloside IV	PTEN, PI3K/AKT, mTOR		Astragaloside IV activates autophagy by miR-21-mediated PTEN/PI3K/AKT/mTOR pathway	(309)
	Supramolecular Nanofibers	TRB3	MRC-5 cells	Supramolecular Nanofibers interfere with aberrant TRB3/p62 PPIs to restore autophagy.	(310)
	Naringin	ATF3, PINK1	C57BL/6 mice	Naringin represses expression of ERS and mitophagy-related genes as well as ERS downstream proteins, thereby activating transcription factor (ATF) 3 and repressing PTEN-induced transcription of kinase 1 (PINK1).	(311)
	Tetrandrine	PINK1, Parkin	MLE-12 cells	Tetrandrine alleviates IPF by inhibiting alveolar epithelial cell senescence through PINK1/Parkin-mediated mitophagy.	(312)
	Zanubrutinib	TGF-β1	Human fibroblasts	It inhibits collagen deposition and myofibroblast activation by inhibiting the TGF-β1/Smad pathway, and induces autophagy through the TGF-β1/mTOR pathway.	(313)
	Bergenin	TGF-β1	Human fibroblasts	Bergenin inhibits myofibroblast activation and promotes autophagy and apoptosis in myofibroblasts.	(314)
	Ellagic Acid	Wnt	Mlg cells, NIH-3T3 cells	Ellagic acid inhibits fibroblast activation and induces autophagy and apoptosis in myofibroblasts mainly by regulating the Wnt pathway.	(315)
	Astragalin	Beclin-1, LC3A/B	BEAS-2B cells	Astragalutin inhibits autophagosome formation in oxidant-exposed airway epithelial cells by reducing beclin-1 and LC3A/B induction.	(316)
	Tubastatin	TGF-β, PI3K/AKT	Human lung tissue samples	Tubastatin ameliorates lung fibrosis by targeting TGFβ-PI3K-Akt pathway via an HDAC6-independent mechanism.	(317)
	Nintedanib	Beclin-1, ATG7, TGF-β	Primary human fibroblasts, IMR-90 cells	Nintedanib inhibits TGF-β signaling, downregulates ECM gene/protein expression, and promotes non-canonical autophagy.	(318)
	Pirfenidone	PARK2, ROS	Human lung tissue	Pirfenidone induces autophagy/mitophagy activation by enhancing PARK2 expression.	(319)
	Dihydroartemisinin	ROS, Fe2 +	HFL1 cells	DHA treatment resulted in Fe2 + deficiency which then triggered ferritin autophagy D in activated HFL1 cells to inhibit fibroblast differentiation into myofibroblasts.	(320)
PH	ROC-325	HIF-1α, HIF-2α, p62	SD rats	ROC-325 exerts vasodilation and antagonizes pulmonary vascular remodeling by inhibiting autophagy, degrading HIF, and activating eNOS-NO signaling.	(321)
	Piperlongumine	LC3B	PASMCs	Piperlongumine may be effective in inhibiting the proliferation of PASMCs by regulating autophagy, reducing pulmonary artery systolic pressure and right ventricular hypertrophy, and improving pulmonary vascular remodeling.	(322)
	Rhodiola crenulata extract	PPARγ, LC3B, ATG7	PAH rats	RCE administration reversed high levels of decadienoyl-L-carnitine by modulating the metabolic enzyme CPT1A in mRNA and protein levels in serum and lung in PAH rats.	(323)
	Metformin	AMPK	PAH rats	Metformin inhibited hypoxia-induced pulmonary vascular remodeling, collagen deposition, pulmonary artery smooth muscle cell proliferation, increased BECN-1 and LC3B-II/-I ratios, and down-regulation of p62 by inhibiting autophagy through activation of the AMPK signaling pathway.	(324)
	Umbelliferone	ROCK	PASMCs	Umbelliferone could ameliorate hypoxia-induced pulmonary hypertension by inhibiting RhoA/ROCK signaling pathway and autophagy.	(325)
	liraglutide	NADPH, NOX	PASMCs	Liraglutide can reduce the proliferation of PASMCs by inhibiting cellular Drp1/nicotinamide adenine dinucleotide phosphate (NADPH) oxidase (NOX) pathway and Atg-5/Atg-7/Beclin-1/LC3β-dependent pathway of autophagy in PAH.	(326)
	Docetaxel	LC3B-II, p62	PAH rats	Docetaxel decreased autophagy as monitored by LC3B-II and p62 expression.	(327)
	Puerarin	LC3B-II, BECN-1, SQSTM1	PASMCs	Puerarin reverses pulmonary vascular remodeling induced by hypoxia in an autophagy-dependent manner.	(328)
	Chloroquine	p62, LC3B-II, BMPR-II	SD rats	Chloroquine prevents the progression of experimental pulmonary hypertension by inhibiting autophagy and lysosomal bone morphogenetic protein type II receptor degradation.	(329)
	Carfilzomib	LC3B-II	SD rats	TP53INP1 specifically drives autophagy to cell death by interacting with LC3B-II in response to Carfilzomib.	(330)
	Quercetin	FOXO1	PASMCs	Quercetin enhanced hypoxia-induced autophagy via the FOXO1-SENS3-mTOR pathway.	(331)
	Apelin	PI3K/Akt, mTOR	PASMCs	Exogenous apelin inhibits autophagy and reduces cell proliferation by activating the APJ receptor-dependent PI3K/Akt/mTOR signaling pathway.	(332)
	Trifluoperazine	AKT	Human pulmonary artery smooth muscle cells	Trifluoperazine reduces activation of the multitasking kinase AKT, resulting in nuclear translocation and slowed proliferation of FOXO3.	(333)
	Iloprost	TGF-β1, LC3B	SD rats	Iloprost significantly induced metalloproteinase-9 gene expression and activity and increased expression of autophagy genes associated with collagen degradation.	(334)
PTB	1,25-dihydroxyvitamin D3	ATG5, Beclin-1	PTB Patient Monocytes	Vitamin D enhances innate immune function, promotes the expression of autophagy-related genes, and may help to control the intracellular growth of Mycobacterium bovis in macrophages.	(335)
	Nordihydroguaiaretic acid	PI3K/AKT, mTOR	THP-1 cells, MDM	Nordihydroguaiaretic acid enhances the immune response by promoting autophagy.	(336)
	MIR144*	DRAM2	ATCC, CRL-3213	MIR144* inhibits the antimicrobial response against Mycobacterium tuberculosis in human monocytes and macrophages by targeting the autophagy protein DRAM2.	(337)
	BTLA	AKT, mTOR	Whole blood of PTB patients, C57/BL6 mice	BTLA promotes host defense against Mycobacterium by enhancing autophagy.	(338)
	Loperamide	LC3, ATG16L1	Balb/c mice	The effect of loperamide on human AM bactericidal activity is associated with degradative autophagy, including ectopic expression and degradation of endogenous p62.	(339)
	Curcumin	NFκB, LC3B	THP-1 human monocytes	Curcumin enhances control of Mycobacterium tuberculosis-infected human macrophages by inducing autophagy.	(340)
NSCLC	Apatinib	ROS/Nrf2/p62 signaling	A549 cells, H1299 cells	Apatinib induces autophagy and apoptosis in NSCLC by modulating ROS/Nrf2/p62 signaling.	(341)
	Andrographolide	p62	H1975cells, H1299cells, H1650cells, H460cells, BEAS-2B cells	Andrographolide regulates P62-dependent selective autophagic degradation of PD-L1 by inhibiting STAT3 phosphorylation.	(342)
	Schizandrin A	p62, AMPK	A549, H1299, H1975, BEAS-2B cells	SchA can induce autophagy by activating the AMPK signal, the autophagy process induced by SchA remains incomplete and fails to promote cell survival.	(343)
	Curcumin	Beclin1, LC3	A549 cells, H1299 cells	Curcumin induces iron death by activating autophagy in NSCLC.	(344)
	Rocaglamide	cGAS-STING, ULK1	A549cells, H1299 cells, H1975 cells	RocA promotes NK cell infiltration by targeting ULK1 to inhibit autophagy.	(345)
	Tryptanthrin	ROS, LC3	A549 cells	Tryptanthrin increases autophagy triggered by the transition from LC3-I to LC3-II to inhibit proliferation and induce apoptosis in non-small cell lung cancer (NSCLC) cells.	(346)
	Polyphyllin VI	ROS, NF-κB	A549 cells, H1299 cells	PPVI-induced caspase-1-mediated pyroptosis in NSCLC by inducing ROS/NF-κB/NLRP3/GSDMD signaling axis	(347)
	Crizotinib	AMPK	CCC-HEH-2	Crizotinib-impaired autophagic process leads to cardiomyocyte death and cardiac injury through inhibition of MET protein degradation, and a novel function of blocking autophagosome-lysosome fusion is demonstrated in drug-induced cardiotoxicity.	(348)
	Tubeimoside-1	mTOR	A549 cells, H157cells, H1299cells, H460 cells, LLC	TBM-1 selectively binds to the mammalian target of rapamycin (mTOR) kinase and suppresses the activation of mTORC1, leading to the nuclear translocation of TFEB and lysosome biogenesis.	(349)
	Ginsenoside Rg5	PI3K/Akt, mTOR	H1650 cells, A549 cells, BEAS-2B cells	Rg5 induces autophagy and caspase-dependent apoptosis in NSCLC cells by inhibiting the PI3K/Akt/mTOR signaling pathway.	(350)
	Resveratrol	AMPK, mTOR	A549 cells	RSV antagonizes NGFR knockdown-induced enhanced autophagy and apoptosis via AMPK, mTOR pathway.	(351)
	Pseudolaric acid B	ROS, AMPK, mTOR	BEAS-2B cells, H1975 cells, H1650 cells	PAB induces apoptosis and autophagic cell death in NSCLC cells via the ROS-triggered AMPK/mTOR signaling pathway.	(352)
	Corynoxine	AKT, mTOR, GSK3β	A549 cells, H1975 cells	Corynoxine triggers cell death by activating PP2A and inhibiting the AKT-mTOR/GSK3β axis.	(353)
	Morusin	JNK, ERK, PI3K/Akt	A549 cells, NCI-H292	Morusin induces apoptosis and autophagy through JNK, ERK and PI3K/Akt signaling in human lung cancer cells.	(354)
	Sophflarine A	ROS	A549cells, H820 cells	Sophflarine A mediates pyroptosis and autophagy via regulating ROS.	(355)
	DFIQ	ROS, LAMP2	H1299cells, A549cells, H460 cells	DFIQ induces ROS production through autophagy activation and LAMP2 depletion.	(356)
	Carnosic Acid	LKB1, AMPK	H1299 cells, H460 cells	CA can induce apoptosis through a mechanism involving sestrin-2/LKB1/AMPK signaling and autophagy induction.	(357)
	Muyin extract	P53, Bcl-2	A549 cells, NCI-H460 cells	Muyin extract induces apoptosis and autophagy by blocking the Akt/mTOR pathway to enhance immunity.	(358)
	ABTL0812	ATF4-DDIT3-TRIB3	A549 cells, MRC5	ABTL0812 inhibits the AKT-mTORC1 axis via upregulation of TRIB3 in cancer cells and tumor models.	(359)
	(+)-anthrabenzoxocinone	PI3K/AKT, mTOR	A549 cells, NCI-H1299, NCI-H226	The PI3K/AKT/mTOR signaling pathway is targeted and suppressed by (+)-ABX, resulting in the induction of S and G2/M phase arrest, apoptosis, and autophagy in NSCLC cells.	(360)
	ASP4132	AMPK	A549cells and NCI-H1944	ASP4132 acts through AMPK activation, mTORC1 inhibition and EGFR-PDGFRα degradation, as well as Akt inhibition and autophagy induction.	(361)
	Baicalein	MAP4K3, mTOR	H1299 cells, A549 cells	Baicalein regulates autophagy in NSCLC via the MAP4K3/mTORC1/TFEB axis.	(362)
	pegaharoline A	PI3K/AKT, mTOR	A549 cells, PC9	PA can inhibit NSCLC cell growth by blocking PI3K/AKT/mTOR and EMT pathways.	(363)
	Ailanthone	ULK1	H1975, A549, HCT-8, MDA-MB-231, BEAS-2B, 293T cells	Ailanthone inhibits ULK1-mediated autophagy and subsequently inhibits NSCLC cells	(364)
	DSTYK	P62, mTOR	H2009 cells, H226 cells	DSTYK inhibits mTORC1, which promotes autophagy, and its defects result in disruption of autophagy, leading to progressive accumulation of autophagosomes.	(365)

### COPD

Based on the available findings, it may be possible to hypothesize that selective targeting of autophagy-related proteins at the genetic or pharmacological level may serve as a basis for the formulation of novel therapies for COPD. In mouse models, many studies have attempted to mitigate the occurrence of abnormal autophagy during smoke exposure by different approaches. However, these investigations have primarily focused on preventive interventions related to the duration of smoking. These studies included the chemical chaperone 4-phenylbutyrate (162); antioxidant drug, cysteine (217); arachidonic acid-derived epoxyeicosatrienoic acid (218); HDAC6 inhibitor tubastatin (219); mitophagy inhibitor Mdivi134; and sodium channel inhibitor carbamazepine. In addition, studies using the mTOR inhibitor rapamycin suggested that increasing autophagy during CS exposure could reduce lung tissue inflammation, which may be of assistance. However, rapamycin increased the number of apoptotic and inflammatory cells compared with controls at baseline. To clarify the pathophysiological function of autophagy in disease, it is essential to carefully time the activation of autophagy and the targeting of lung cells. Further investigation is required to assess the impact of these agents on dysregulated autophagy in COPD.

### CF

Treatments for CF have been extensively investigated. The restoration of autophagic functionality may provide additional therapeutic options for treating CF. The antioxidant n-acetyl-l-cysteine has been shown to improve airway phenotypes in CFTR mutant mice. In addition, oral cysteamine was found to restore Beclin 1 expression and prolonged the survival of CFTR ^F508del^ mutant mice (220). Hence, it may be worthwhile to investigate cysteamine drugs’ mechanism through restoring autophagy (221). In addition, regular and continuous use of azithromycin has been demonstrated to enhance the health condition of CF patients (222, 223). However, It has been reported that mycobacterial infection increases synchronously with the onset of CF in some studies (224, 225). The seemingly contradictory results observed in cystic fibrosis patients treated with azithromycin may be attributed to the drug’s capacity to inhibit lysosomal acidification. This inhibition subsequently disrupts autophagy and the degradation processes within phagosomes. More therapeutic options targeting autophagy-related pathways need to be investigated in greater depth.

### IPF

Promoting autophagy may be beneficial in the treatment of IPF. Currently, drugs that may be active include IL17A neutralizing antibodies (177), MIR449A(microRNA 449a) (226), or PDGFRB (platelet-derived growth factor receptor beta) inhibitors. Bleomycin-mediated increases in mortality and decreases in fibrotic resistance in mice have been observed in experimental models (178, 227). However, rapamycin appears to potentiate silica-induced effects, exacerbating inflammation and fibrosis (228). In individuals diagnosed with IPF, the rapamycin analog everolimus has been observed to contribute to the progression of the disease. Therefore, further studies are required to assess whether targeted autophagy agents are beneficial in IPF.

### Pulmonary hypertension

Numerous studies utilizing animal models have investigated the impact of rapamycin, mTOR inhibitors, and autophagy activators on the prevention of PAH development (229). In clinical settings, everolimus, a derivative of rapamycin, has been shown to enhance outcomes in patients with strict PH resulting from chronic thromboembolic disease, while also decreasing pulmonary vascular resistance (230). The suppression of the autophagy pathway may offer potential targeted therapeutic strategies for the disease.

### Pulmonary tuberculosis

Therapeutic regimens targeting pulmonary tuberculosis by autophagy-related pathways are being widely investigated. Vitamin D stimulates autophagy activation in Mycobacterium tuberculosis by inducing antibiotics (231). Vitamin D deficiency has been linked to a heightened threat of active TB (232). The conversion time of sputum cultures is not affected by vitamin D supplementation according to recent research (233, 234). However, it has also been reported that vitamin D supplementation given to patients with vitamin D receptor polymorphisms shortens sputum culture conversion time (234). Isoniazid and pyrazinamide are recognized as primary agents in the treatment of tuberculosis. These compounds facilitate the activation of autophagy and the maturation of autophagosomes within host cells infected by Mtb (235). This may constitute a component of the fundamental mechanism associated with the treatment involving these agents. Although the activation of autophagy has been previously proposed as a viable therapeutic approach for patients infected with Mtb, recent findings cast uncertainty on this hypothesis and impede the progression of further research.

### Non-small cell lung cancer

The development of novel compounds aimed at targeting mutant p53 and reinstating its wild-type functionality represents a promising therapeutic approach for cancer treatment, particularly in the context of NSCLC, which is characterized by a significant mutation frequency (236–238). This therapeutic potential has already been demonstrated in many compounds. Nutlins are cis-imidazoline analogs that inhibit the interaction between MDM2 and wild-type p53 *in vivo*, which in turn enhances the anti-tumor ability of p53 (239). We speculate that the development of targeted agents against aberrant p53 or promoting anti-tumor activity of wild-type p53 may be helpful in the treatment of cancer. RETRA was found to inhibit the malignant proliferation of cancer cells carrying aberrant p53 via a p73-dependent salvage pathway (240). The reactivating small molecule PRIMA-1 of mutant p53 can be combined to convert it into a wild-type construct, thereby achieving inhibition of tumor growth (241, 242). In addition, restoring and stabilizing the DNA binding domain (DBD) of p53 is also a promising tumor suppressor strategy.

Rapamycin has great potential in cancer therapy, which activates mitochondria-mediated apoptosis independent of p53 in NSCLC cells, stressing its effectiveness in disease (29). It has been shown that mouse models of NSCLC have reduced tumor growth and apoptosis following rapamycin treatment (232). Furthermore, certain anticancer agents have demonstrated markedly enhanced efficacy when administered in conjunction with rapamycin. This includes Bcl-2 inhibitors such as ABT-737, pemetrexed, and lipophilic bisphosphonates (196). Additionally, EGFR tyrosine kinase inhibitors (TKIs) have received approval for the treatment of patients with NSCLC who possess particular EGFR mutations (243). However, resistance to this drug is a major problem in clinical treatment. Notably, erlotinib combined with rapamycin enhanced autophagy and restored sensitivity to EGFR-TKIs (244). Erlotinib in combination with rapamycin has also been shown to help overcome resistance due to p53 deficiency *in vitro*.

In addition to rapamycin, drugs targeting proteins related to other signaling pathways of autophagy, such as AZD8055 (PI3K inhibitor), NVP-BEZ235 (PI3K and mTORC1 inhibitors), perifosine (AKT inhibitor), and GSK-690693 (AKT inhibitor), have been investigated in NSCLC. In certain instances, these targeted therapies modulate autophagy-related pathways as a component of the treatment regimen for NSCLC (245–248). The effects of some traditional Chinese medicine compounds in NSCLC have also been largely investigated. Curcumin is a phenolic compound derived from the plant Curcuma longa (249, 250). Curcumin treatment showed a promoting effect of autophagy as well as a pro-apoptotic effect in lung adenocarcinoma A549 cells, allowing us to speculate its therapeutic potential in NSCLC (251). Cytoprotective autophagy in NSCLC cells is also activated by cucurbitacin E and glycerinic acid (252). Licochalcone A, a flavonoid derived from the traditional Chinese medicinal plant Glycyrrhiza uralensis Fisch, has been shown to promote apoptosis and autophagy through the induction of ER stress (253). Thick acid from Poria cocos halted lung cancer cell growth by boosting ROS and activating JNK (254). Platycodin-D can induce autophagy in H460 and A549 NSCLC cells, as shown by stimulating the formation of ATG3, ATG7, Beclin-1, and LC3-II (255).

## Conclusion

Autophagy plays a dual role in lung diseases, exhibiting both potentially harmful effects in certain pathophysiological conditions and serving as a protective mechanism that promotes cell survival. Recent advancements in research have significantly enhanced our understanding of the role of autophagy in the pathophysiological mechanisms underlying various diseases, thereby offering novel insights for the development of targeted therapies for pulmonary diseases. However, assessing the actual clinical effects of targeted agents for autophagy-related pathways on lung diseases is challenging. The challenge arises from the observation that autophagic responses occurring in various compartments of the lung may yield markedly distinct effects. Accurate measurements of autophagy also need to be updated. It is critical to revise the precise assessments of autophagy. Understanding the impact of highly specific autophagy modulators on disease models characterized by particular autophagy deficiencies is vital for formulating clinically relevant approaches to either stimulate or suppress autophagy.
